# Imprinted Gene Expression and Function of the Dopa Decarboxylase Gene in the Developing Heart

**DOI:** 10.3389/fcell.2021.676543

**Published:** 2021-06-22

**Authors:** Adam R. Prickett, Bertille Montibus, Nikolaos Barkas, Samuele M. Amante, Maurício M. Franco, Michael Cowley, William Puszyk, Matthew F. Shannon, Melita D. Irving, Marta Madon-Simon, Andrew Ward, Reiner Schulz, H. Scott Baldwin, Rebecca J. Oakey

**Affiliations:** ^1^Department of Medical and Molecular Genetics, King’s College London, London, United Kingdom; ^2^Department of Clinical Genetics, Guy’s and St Thomas’ NHS Foundation Trust, London, United Kingdom; ^3^Department of Biology and Biochemistry and Centre for Regenerative Medicine, University of Bath, Bath, United Kingdom; ^4^Department of Pediatrics (Cardiology), Vanderbilt University Medical Center, Nashville, TN, United States

**Keywords:** dopa decarboxylase, knock-out, imprinting, heart, mouse, human

## Abstract

Dopa decarboxylase (DDC) synthesizes serotonin in the developing mouse heart where it is encoded by *Ddc_exon1a*, a tissue-specific paternally expressed imprinted gene. *Ddc_exon1a* shares an imprinting control region (ICR) with the imprinted, maternally expressed (outside of the central nervous system) *Grb10* gene on mouse chromosome 11, but little else is known about the tissue-specific imprinted expression of *Ddc_exon1a*. Fluorescent immunostaining localizes DDC to the developing myocardium in the pre-natal mouse heart, in a region susceptible to abnormal development and implicated in congenital heart defects in human. *Ddc_exon1a* and *Grb10* are not co-expressed in heart nor in brain where *Grb10* is also paternally expressed, despite sharing an ICR, indicating they are mechanistically linked by their shared ICR but not by *Grb10* gene expression. Evidence from a *Ddc_exon1a* gene knockout mouse model suggests that it mediates the growth of the developing myocardium and a thinning of the myocardium is observed in a small number of mutant mice examined, with changes in gene expression detected by microarray analysis. Comparative studies in the human developing heart reveal a paternal expression bias with polymorphic imprinting patterns between individual human hearts at *DDC_EXON1a*, a finding consistent with other imprinted genes in human.

## Introduction

There are over a hundred imprinted genes in the mouse and human genome (Kelsey and Bartolomei, 2012) many of which contribute to mammalian growth and development (Cleaton et al., 2014). Genomic imprinting is the parent-of-origin-dependent, allele-specific expression of a gene (Ideraabdullah et al., 2008). Imprinted genes are regulated by epigenetic mechanisms including parent-of-origin-dependent, allele-specific DNA methylation of CpG-rich differentially methylated regions (DMRs). There are two classes of DMRs: germline DMRs that are established during gametogenesis are maintained throughout development and act as imprinting control regions (ICRs); and somatic DMRs that arise post-fertilization, are often tissue-specific and can contribute to the regulation of imprinted gene clusters together with the ICR (Edwards and Ferguson-Smith, 2007). Both classes are maintained during cell division (Lewis and Reik, 2006).

The Dopa decarboxylase gene (*Ddc)* has two transcript isoforms, one is expressed from both parental alleles and the other is imprinted. *Ddc*, is expressed from both parental alleles in the urinary system, eye, nervous system, liver, limbs, alimentary system and ear. Deficiency of this canonical form of the gene in humans results in an autosomal recessive inborn error of metabolism (MIM #608643) (Lee et al., 2013; Smith et al., 2014). The second transcript isoform, known as *Ddc_exon1a* was identified through the analysis of a differential gene expression screen designed to detect novel imprinted genes and it is imprinted, being expressed only from the paternally inherited allele in the developing mouse heart (Menheniott et al., 2008).

Imprinted genes are typically found in clusters in the genome (Barlow and Bartolomei, 2014) and *Ddc_exon1a* is no exception lying adjacent to *Grb10*, an imprinted gene that encodes an intracellular signaling adaptor protein. *Grb10* is typically maternally expressed (Charalambous et al., 2003) and acts to restrict fetal growth and promote adipose deposition in adulthood (Smith et al., 2007; Madon-Simon et al., 2014). Unusually, *Grb10* is expressed from the opposite (paternal) allele in the CNS but the mechanism that underlies this switch between maternal and paternal expression is unclear, as is the role for paternally expressed *Grb10* in neurons (Plasschaert and Bartolomei, 2015). The two genes therefore comprise an imprinting cluster where imprinted expression is directed via the shared ICR in the 5′ untranslated region (UTR) of *Grb10* [also known as the *Grb10* CpG island 2 (CGI 2) DMR]. When the ICR is ablated, it results in loss of both *Grb10* and *Ddc_exon1a* imprinting (Shiura et al., 2009). Since the regulation of imprinted gene clusters is typically co-ordinated (Ideraabdullah et al., 2008), we reasoned that *Ddc_exon1a* expression could be co-ordinately regulated with the expression of *Grb10*.

*Ddc_exon1a* is the only variant of Ddc expressed in heart and is a unique example of a transcript that shows heart-specific genomic imprinting. *Grb10* has a more complex imprinted expression pattern in the developing embryo but exhibits paternal expression in the CNS. There are varying reports regarding *Ddc_exon1a* expression in the brain (Shiura et al., 2009; Madon-Simon et al., 2014; Smith et al., 2014; Plasschaert and Bartolomei, 2015). As *Grb10* is expressed maternally in most tissues but shows paternal expression specifically in the brain and in subsets of cells in the heart, this suggested to us that *Ddc_exon1A* and *Grb10* might be linked and coordinately expressed in brain and heart.

We sought to investigate the allelic expression of *Ddc_exon1a* in the brain and the heart using allele-specific assays in tissues from reciprocal hybrid mouse strains. As allelic expression was predominantly observed in the heart, the spatial pattern of DDC_EXON1A protein was delineated in the developing mouse heart by immunostaining embryonic sections. Spatial distribution was also compared to its ICR partner *Grb10* in a gene trap transgenic mouse line. To investigate function, the phenotype of a knockout mouse model of *Ddc_exon1a* was examined and changes in the developing myocardium were seen along with gene expression changes associated with tissue development and cellular organization. An antisense transcript overlaps *Ddc_exon1a* but no evidence was found for it influencing imprinted *Ddc_exon1a* expression *in cis*. A comparative study examining the expression of *DDC_EXON1A* in 40 human fetal hearts including fetal-maternal pairs, reveals a paternal expression bias and a polymorphic pattern of imprinted gene expression.

## Materials and Methods

### Allele-Specific RT-PCR Assays

RNA was extracted from tissue using the RNAeasy Kit^TM^ (Qiagen), assessed for purity using NanoDrop (requiring a 260/280 ratio of ∼2.0) and integrity using Agilent 2100 Bioanalyzer, and converted to cDNA with a Superscript II^TM^ (Invitrogen) kit, as per manufacturers’ instructions. For mouse *Ddc_exon1a* the allele was identified via a G/A single nucleotide polymorphism (SNP) between *Mus musculus domesticus* C57BL/6J (B) and *Mus musculus castaneus* CAST/EiJ (C) in exon 3 (mm9, chr11:11776278). Transcripts with this SNP were amplified by PCR from reciprocal BxC and CxB hybrids (by convention, the maternal genotype is listed first) and sequenced. *Ddc_exon1a* and *Ddc_canonical* transcripts were amplified using exon-specific forward primers EXON1A-F (5′-TGTCACCAAGGAGAGAGAGAGA-3′) and EXON1-F (5′- AGTTGTGTCGCCACCTCCT-3′) and a common reverse primer, EXON4-R (5′-CAGCTCTTCCAGCCAAAAAG-3′). PCR: 94°C for 3 min, 34 cycles of 94°C for 30 s, 56°C for 30 s, and 72°C for 1 min, with a final extension of 72°C for 5 min and Sanger sequencing using an ABI 3730xl.

For AK0066690, nested primers were used AK006690_ F_outer: CCAGCCTCCATTTCAGAGTT, AK006690_R_outer: TTGACTAGGAATATTTCCTTCCAT, amplicon size: 250bp. Inner primers were AK006690_F_inner: TTCAGCCAAGAG TGCTTAGG, AK006690_R_inner: GCTGCTGCATGCTTAT TTGT, amplicon size: 184 bp.

### Immunostaining

E15.5 wildtype embryos were fixed in 4%PFA for 1 h at 4°C, dehydrated and embedded in paraffin wax. Antigen retrieval was performed by boiling in high pH antigen unmasking solution (Vector Labs). Slides were blocked with 4% v/v donkey serum (abcam, ab7475) for 1.5 h. Primary antibodies in the following dilutions: anti-DOPA Decarboxylase antibody (ab3905) (1:500), goat-α-mouse ANF (1:100) in 0.01% Tween-20, 2% v/v donkey serum in PBS were dropped onto slides and incubated in a humidified chamber at 4°C for 16 h. Slides were washed 3X in 0.01% Tween-20 in PBS. Secondary antibodies Alexa Fluor 555 donkey-α-goat (Invitrogen) and Alexa Fluor 647 donkey-α-rabbit (Invitrogen) were diluted 1:300 in 0.01% Tween-20 PBS, dropped onto slides and incubated for 2 h at RT in the dark. Slides were washed 3X in PBS and mounted using ProLong^®^ Gold Antifade Reagent with DAPI (Invitrogen).

### Histological Analysis

Histological analysis was performed on the *Grb10KO* with a *lacZ* reporter construct at the 3′ end of exon 8 (Garfield et al., 2011). Gestating Grb10KO females were sacrificed at 18.5 days *post coitum* and the uterine horns isolated immediately. All animal experiments were approved and regulated by the university ethics committee at the University of Bath and conform to the guidelines from Directive 2010/63/EU of the European Parliament on the protection of animals used for scientific purposes. The reporter insertion ablates all isoforms of *Grb10* in mouse embryos and results in a null. Where *Grb10* expression is perturbed, lacZ protein expression occurs. In *Grb10^+/KO^* mice, tissue localization of *Grb10* is blue with X-gal staining. *Grb10^+/KO^* E15.5 embryos were fixed in 4% (w/v) PFA, cryoprotected in 30% sucrose and embedded in OCT. Sections were stained for paternal *Grb10* in 1 mg/ml X-gal diluted in stain base [30 mM K_4_Fe(CN)_6_ 30 mM K_3_Fe(CN)_6_.3H_2_O, 2 mM MgCl_2_, 0.01% (w/v) sodium deoxycholate, 0.02% (v/v) Igepal CA-630 in 0.1% PBS] for 18 h at 28°C and counterstained using nuclear fast red.

Samples were stained for DDC using VECTASTAIN^®^ ABC kit (Vector Labs) with blocking in 5% skim milk. DDC antibody (Abcam, #3905) was used at a 1:500 dilution. Sections were counterstained with Harris’ hematoxylin (30 s), and incubation in Scott’s tap water (Fisher) for 1 min.

### Morphological Analysis Using Episcopic Fluorescence Image Capture (EFIC)

Embryos and dissected neonatal mouse hearts were fixed and embedded in paraffin as for immunostaining. Measurements of embryos were adjusted to the crown rump length to account for differences in embryo size due to variation in the time of conception on a given day of gestation as is convention. For EFIC analysis (Rosenthal et al., 2004) sections were re-embedded in red aniline dyed wax and photographed using an EFIC system at Vanderbilt University, with a sectioning size of 5μm. Samples were photographed at a magnification of 20× using appropriate mCherry and GFP wavelengths. Images were quality controlled by visual inspection and rebuilt in 3D using Volocity^TM^ image analysis software (Perkin Elmer) and virtually re-sectioned in a plane that bisected the mitral and aortic valve, with the measurements taken on this plane at the base of the papillary muscle to ensure samples were measured equally. All measurements were made blind with the identity of the samples only revealed prior to statistical analysis. Comparisons between sample groups were made using a Mann-Whitney test.

### Microarray Analysis

Microarray libraries were generated as per manufacturers’ instructions for Affymetrix Genechips^TM^ on three *Ddc*^*WT*^ and four maternally deleted (*Ddc^*MAT*Δ^*) (making seven wild type samples) and four paternally deleted (*Ddc^*PAT*Δ^*) and one homozygous mutant (*Ddc^ΔΔ^*) (providing five knockouts of *Ddc_exon1a* in embryonic heart) using two separate six-lane arrays. Raw probe signals were background-corrected using NEQC quartile-normalized and a linear model was fitted to compare the effects of different genotypes in LIMMA (Smyth, 2004). These data have been deposited in GEO, Accession number GSE87595.

### Western Blotting

An E15.5 embryo carcass was macerated in 1 ml of RIPA buffer [50 mM Tris-HCl (pH 7.5), 150 mM NaCl, 1 mM EDTA, 1% (w/v) sodium deoxycholate, 0.1% SDS, 1 mM PMSF, 1× protease inhibitor (Roche)] and centrifuged at 16,000 g for 20 min at 4°C. Total protein in the supernatant was measured using the BCA protein assay kit (Pierce) and stored at −20°C. The same protocol was used to extract protein from cultured cells without maceration. 20 μg in 25 μl of each sample was mixed 1:1 with 2× reducing buffer (62.5 mM Tris HCl pH 6.8, 2% (w/v) SDS, 6 M Urea, 2% (v/v) Igepal CA-630, 5% (v/v) β-mercaptoethanol, 0.02% (w/v) bromophenol blue, 4% glycerol and heated to 95°C for 5 min. Samples were electrophoresed alongside a Spectra^TM^ multicolor protein ladder (Thermo Fisher Scientific) on a 12% polyacrylamide resolving gel: 12% polyacrylamide (National Diagnostics), 0.37M Tris:HCl pH 8.8, 0.1% SDS, 0.05% AMPS, 0.05% TEMED with a stacking gel (5% polyacrylamide, 0.12M Tris:HCl pH 6.8, 0.05% AMPS, 0.1% TEMED) at 100 V for 3 h in running buffer (0.1% SDS, 25 mM Tris, 208 mM glycine). Protein was transferred at 90 V for 2 h to a PVDF membrane (Bio-Rad) using western blot wet transfer buffer (25 mM Tris, 192 mM glycine, 20% (v/v) methanol. The membrane was blocked for 90 min in 5% powdered skimmed milk (Marvel) in 0.1% Tween-20 with PBS. Primary antibodies were diluted, rabbit-α-mouse DDC (1:1,000) in 5% milk in 0.1% Tween-20 with PBS and incubated with the membrane overnight at 4°C. Membranes were washed 3X in 0.1% Tween-20 with PBS for 15 min and incubated in peroxidase-conjugated goat anti-rabbit secondary antibody (DAKO P0448) diluted 1:2,000 in 5% milk in 0.1% Tween-20 with PBS for 1 h at RT. 3X washes were performed and protein detected using the ECL system (Amersham). Proteins were visualized by exposure to Fuji film developed on a Laser45 machine. For loading control, membranes were stripped by heating at 50°C for 30 min in stripping buffer (100 mM 2-Mecaptoethanol, 2% SDS, 62.5 mM Tris:HCl, pH6.7), washed 3X in 0.1% Tween-20 with PBS for 15 min, and re-probed using mouse-α-mouse Tubulin (abcam anti-alpha Tubulin antibody ab7291; 1:5,000) and rabbit-α-mouse Histone H3 (abcam H3 ab1791; 1:5,000).

### Knockout Mice

*Ddc* knockout model generation was carried out by Lexicon Genetics Inc., United States and breeding, genotyping and tissue acquisition by the UC Davis mouse biology program. The mouse strain and cell lines are deposited as frozen embryos in the International Mouse Strain Resource^1^ and listed in MGI as Ddc^Gt(OST129277)Lex^ (B6;129S5-^DdcGt(neo)420Lex^, ID# 11693–UCD). Homozygous null mice have a lethal phenotype and their number is lower than Mendelian expectations at E10.5 (for example, four heterozygous inter-crosses of this knock-out mouse resulted in 11 WT, 14 Heterozygous and 3 double knock-out embryos. Mendelian ratios would have expected numbers in line with 11 WT, 22, Heterozygous and 11 double knock-out embryos) non-Mendelian ratios were also observed at litters dissected at E15.5. Heterozygous mice did not show overt phenotypes in the Lexicon Genetics high-throughput phenotype screen which aimed to identify genes that when ablated, resulted in overt phenotypes in obesity and skeletal anomalies. This screen is acknowledged to be conflicted between studying individual lines of mice and screening many lines rapidly. Therefore, compromises were made in terms of phenotypic detail, making detailed analyses of heterozygotes essential at the individual gene/strain level. Here we examine the embryos in detail for cardiac phenotypes which were not scored in the Lexicon Genetics screen. The Grb10KO knockout mouse strain was that described in Garfield et al. (2011).

### Human Tissue Acquisition

Twenty-five human heart and matched decidua from 4 to 13 weeks were provided by the MRC-Wellcome Trust Human Developmental Biology Resource (HDBR)^2^ from the Institute of Genetic Medicine, Newcastle and Institute of Child Health, London. Fifteen fetal heart samples and matched maternal cheek swabs were collected via an approved protocol from the Joint Research Ethics Committee of London, Camberwell St Giles, project ID 53717. Informed consent was obtained for the inclusion of these samples. The study was performed abiding by the ethical principles underlying the Declaration of Helsinki and good practice guidelines on the proper conduct of research.

**Human embryo allele-specific assays** by RT-PCR and Sanger sequencing. RNA was extracted from tissue and converted to cDNA using the RNAeasy^TM^ (Qiagen) and Superscript II^TM^ (Invitrogen) kits, as per manufacturers’ instructions. gDNA was extracted using a DNAeasy kit^TM^ (Qiagen). SNPs were identified in the UCSC genome browser and amplified with primers:

DDC_13/15R:GGCATTTAGCCACATGACAA–59.5
DDC_13/15F:ATTCTGGGGCTTGTCTGCTT–61.2
DDC_1/4F:TGGAGAATCCCATCAAGGAG–60.0
DDC_1/1a/4R:CACAGTCTCCAGCTCTGTGC–59.8
DDC_1a/4F:GGACAGAGAGCAAGTCACTCC–59.0
DDC_1a/4F2:CTGTCACTGTGGAGAGGAGAG–57.6 and sequenced on an ABI 3730xl.

## Results

### Ddc-Exon1a Is Predominantly Found in the Developing Mouse Heart

**FIGURE 1:**
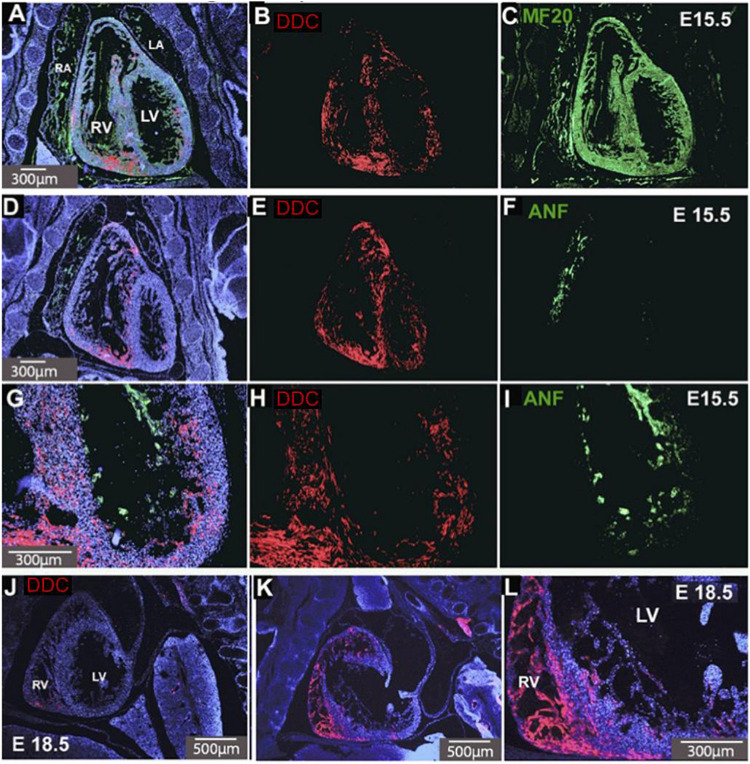
DDC in E15.5 and E18.5 hearts. Coronal sections of the heart co-stained for DDC (red) and the myocardial marker MF-20 (green) **(A–C)** and DDC and a marker of trabeculae, ANF (green) **(D–I)**. DDC protein (red) is present throughout the ventricular myocardium and interventricular septum (IVS), the structure separating the right and left ventricles, **(B,E,H)**, but absent from the atria and the trabecular layer, except where the trabeculae meet the compact myocardial layer **(D–I)**. This is also seen in sagittal sections at E18.5 **(J–L)** where DDC is present in the myocardium, IVS, and right ventricular myocardium. Blue staining is DAPI nuclear counterstain **(A,D,G,J,K,L)**. RV Right Ventricle, LV Left Ventricle, RA Right Atrium, LA Left Atrium. Minimum number of hearts examined = 3.

Fluorescence immunostaining reveals the cellular distribution of cardiac DDC protein in E15.5 hearts. Because *Ddc-Exon1a* is the only isoform expressed in the developing heart (Menheniott et al., 2008) the staining reflects its expression. Sections in the coronal plane show a four-chamber view of the heart (Figure 1) which were either co-stained for DDC and MF-20 (an antibody to the myocardial marker myosin heavy chain) (Figures 1A,C), or DDC and ANF (a marker of trabeculae, the complex meshwork of myocardial strands) (Figures 1D–I). DDC protein was present throughout the ventricular myocardium and inter-ventricular septum (IVS), the structure between the right and left ventricles (Figures 1B,E,H), but was absent from the atria and the trabecular layer, except where the trabeculae meet the compact myocardial layer (Figures 1D–I). In addition, DDC protein was not detected in the epicardium or endocardium. Expression in cardiac fibroblasts is not ruled out, but this cell population is a small component of the ventricular wall at this stage of development (Lajiness and Conway, 2014). Scattered DDC is detected throughout the compact myocardium of both ventricles, with protein present in the myocytes of the right ventricular apex, outflow tract, and right ventricular portion of the interventricular septum at E15.5, and this same pattern was observed at E18.5 (Figures 1E,J–L). All of these regions of the myocardium (RV apex, RV outflow tract, and RV portion of the interventricular septum) are derived primarily from progenitor cells of the secondary heart field and are particularly susceptible to abnormal development leading to congenital heart defects (Kelly, 2012).

**FIGURE 2:**
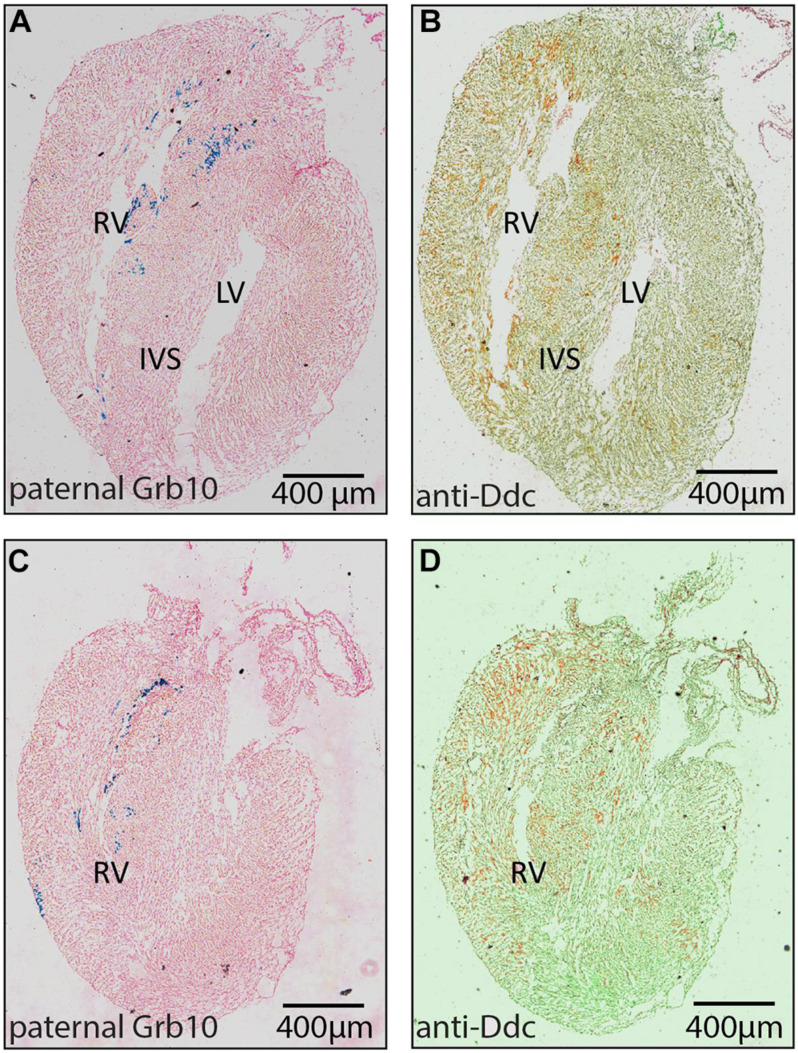
Analysis of paternal *Grb10* expression and DDC protein staining in E18.5 heart serial sections. **(A,B)** are serial sections in the sagittal plane showing the right ventricle (RV) and the left ventricle (LV). **(C,D)** are serial sections showing only the RV where DDC staining is most abundant. **(A,C)** are stained with X-gal to reveal paternal *Grb10* expression in blue from the lacZ reporter construct and the counterstaining is pink. **(B,D)** are stained for DDC protein using DAB (brown) via immunohistochemistry, counterstaining is pale green. DDC protein distribution is extensive compared to *Grb10* which is restricted to the inter ventricular septum (IVS). Minimum number of hearts examined = 3, maximum number = 6.

*Ddc_exon1a* exists in an imprinting cluster along with *Grb10* and their imprinted expression is coordinated by the *Grb10* ICR. Paternal *Grb10*, which is expressed in a subset of cells in the heart, was detected using a *Grb10-*LacZ reporter transmitted through the paternal germline (Garfield et al., 2011). Its restricted expression appears punctate in the IVS and the atrio-ventricular septum (Figure 2) and is present in a small region next to the right ventricular myocardium suggestive of a coronary vessel. Paternally derived DDC_EXON1a protein, however, was more broadly evident throughout the myocardium (Figure 2). DDC_EXON1a protein and *Grb10* gene expression assays do not provide a direct comparison, but these data indicate that DDC_EXON1a and *Grb10* are not obviously present in the same cell types by visual inspection, therefore these genes are not likely to share tissue-specific regulatory elements.

*Ddc_exon1a* expression was also examined in whole brain (Supplementary Figure 1), because a number of imprinted genes exhibit tissue-specific imprinting in the brain (Ferrón et al., 2011) and because of the known switch to paternal expression of *Grb10* in neuronal cells. The assay may, however, be limited because the glia present in whole brain tissue samples express *Grb10* reciprocally from the maternal allele, which could confound an allelic-specific assay of mixed cell types (Yamasaki-Ishizaki et al., 2007). Allele-specific assays measure the height of sequencing peaks from parental alleles and here indicate that *Ddc_exon1a* is expressed from both parental alleles in whole brain, and in some sub-regions including the pre-optic area of the hypothalamus, the cerebellum and the brain stem (Supplementary Figure 1) consistent with published studies (Gregg et al., 2010a,b; DeVeale et al., 2012). We therefore examined data from a single cell type to complement this analysis. Transcriptomic data analysis in neural stem cells from C57Bl/6J × JF1 hybrids (Bouschet et al., 2017) can be utilized to assay allele-specificity by counting the number of aligned sequencing reads originating from each parental allele using SNPs between the two mouse strains. In neural stem cells there is low expression of *Ddc*, but a slight paternal bias of expression is detected at a number of different SNPs (albeit in the common part of the gene with *Ddc* canonical) (Supplementary Figures 1C,D).

Antisense transcripts are involved in imprinted gene regulation at several well characterized loci (Sleutels et al., 2002; Redrup et al., 2009). The *AK006690* transcript at this locus is annotated as transcribed in the antisense direction to *Ddc_exon1a* and its expression was confirmed in newborn brain, heart and liver (data not shown). *AK006690* was assayed for allele-specific expression in heart and brain at E13.5, E16.5/E15.5 and newborn stages in mouse reciprocal hybrids. The transcript was found to be expressed from both parental alleles in brain and heart at E13.5 with a bias toward expression from the paternal allele in later stages of development in heart (Supplementary Figure 2). The observed allelic expression bias also can have a genetic cause, for example the influence of a nearby SNP on the amplification efficiency, but reciprocal hybrid assays suggest that the parental expression in heart at later stages is from the paternal allele (Supplementary Figure 2).

### Characterization of a *Ddc_exon1a* Deleted Mouse Model

*Ddc_exon1a* is expressed from the paternal allele in developing heart (Menheniott et al., 2008) therefore mice inheriting a null allele through the paternal line do not express *Ddc_exon1a* in this tissue. Four genotypes were assayed for expression; *Ddc^*WT*^, Ddc^*MAT*Δ^* (maternal deletion), *Ddc^*PAT*Δ^* (paternal deletion), and *Ddc^ΔΔ^* (deletion on both alleles). Quantitative PCR showed diminished expression in *Ddc^*PAT*Δ^* compared to *Ddc*^*WT*^ and *Ddc^*MAT*Δ^* embryos (Supplementary Figure 3). There is a minor contribution from the maternal allele in the *Ddc^*PAT*Δ^* genotype with the majority being derived from the paternal allele (Supplementary Figure 3), an observation that is consistent with imprinted gene expression (Horsthemke et al., 2011; Korostowski et al., 2011). DDC protein was not detected in *Ddc^ΔΔ^* animals (Supplementary Figure 3).

**FIGURE 3:**
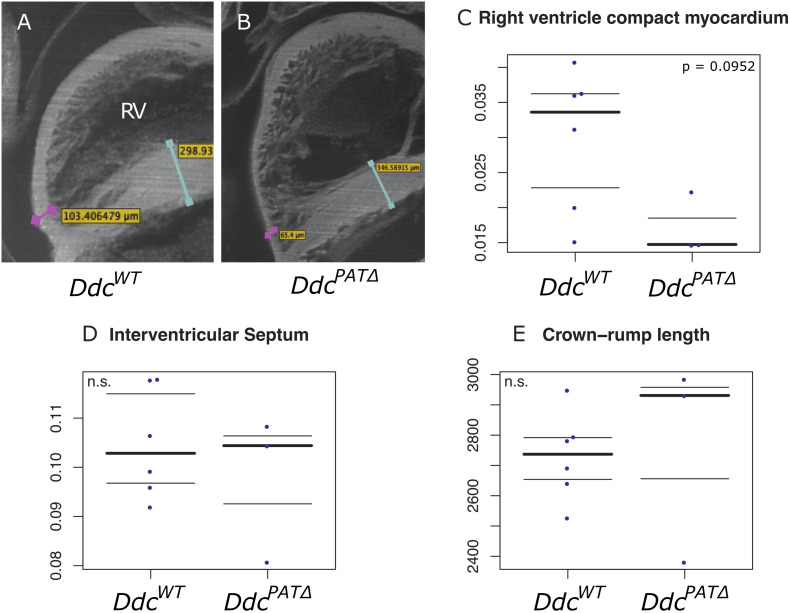
Morphological analysis of *Ddc* knockout hearts. Episcopic fluorescence image capture measurements of wildtype *Ddc*^*WT*^ and *Ddc^*PAT*Δ^* knockout E15.5 mouse heart regions using embedded, sequentially sectioned hearts built into a 3D model using the Velocity^TM^ software. Visualization of the 3D ventricles depicting where the measurements were made in **(A)** *Ddc*^*WT*^ and **(B)** *Ddc^*PAT*Δ^* embryos. The thickness of the right ventricle (RV) compact myocardium at the apical point parallel to the interventricular septum (IVS) is shown by the pink bar in **(A)** and pink bar in **(B)**. The IVS measured at the widest point is indicated by the turquoise bar in **(A)** and the turquoise bar in **(B)**. All measurements were adjusted to crown rump length to control for embryo size variation **(C–E)**. A thinning of the compact layer in the right ventricle is suggested **(C)** in knockout animal hearts compared to wild type with no change in the width of the IVS **(D)** or crown-rump length **(E)** but the total numbers of embryos studied was not sufficient to show statistical significance.

Morphological changes at key sites of cardiac *Ddc* expression, including the width of the IVS and the thickness of the compact layer at the apical region of the right ventricle were measured in the *Ddc^*PAT*Δ^* heart. An episcopic system (Rosenthal et al., 2004) was used to eliminate distortions associated with sectioning at E15.5 in hearts from *Ddc*^*WT*^ (*n* = 6), and *Ddc^*PAT*Δ^* (*n* = 3) animals which were all scored blind to the genotype. *Ddc^*PAT*Δ^* embryos had a thinner compact layer in the right ventricle compared to *Ddc*^*WT*^ by 0.019 μm (Figure 3). A Mann-Whitney test did not reveal a difference in these measurements at a significance level of 5 % (*p* = 0.0952) with the number of mice examined but does suggest the possibility of thinning in the mutants. Thinning of the RV compact layer in the three ablated three ablated embryos could be the result of several different mechanisms (decreased myocyte proliferation, increased myocyte apoptosis, decreased progenitor cell expansion in the secondary heart field, or altered endocardial myocardial interactions) and further mechanistic resolution would be useful but is beyond the scope of this study. No statistically significant differences between *Ddc*^*WT*^ and *Ddc^*PAT*Δ^* were observed for the septum thickness or overall embryo size measured by crown-rump length again measured blind to genotype.

Expression microarrays were performed between *Ddc*^*WT*^ and Ddc*^ΔΔ^* mice and the major difference detected was the *Ddc* gene itself (Supplementary Table 1). The modest impact on the transcriptome might be because the samples were heterogeneous or because the *Ddc*^*WT*^ and *Ddc^*MAT*Δ^* were combined as were the *Ddc^*PAT*Δ^ and* Ddc*^ΔΔ^*. Perturbations in molecular pathways could explain associated phenotypes and the ontology analysis (Supplementary Table 1) supports a role for DDC in cardiomyocyte growth and proliferation.

### Imprinted Expression of *DDC_EXON1A* in Human Heart Tissues

**FIGURE 4:**
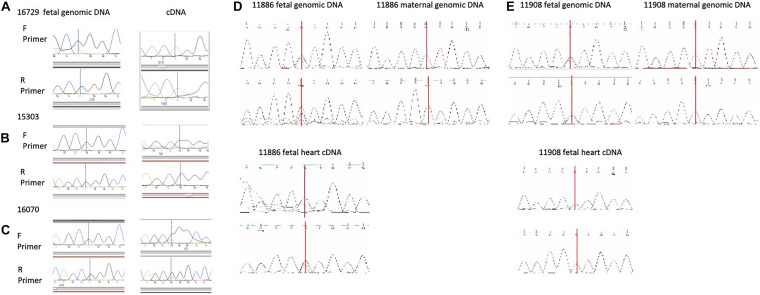
**(A,B)** Human fetal heart allele-specific assays, informative samples from 25 hearts collected from a tissue bank. Individuals in **(A,B)** are mono allelic (one peak across the SNP) and the individual in **(C)** presented with a biallelic pattern of expression (2 peaks across the SNP). SNPs annotated in the UCSC genome browser were identified in these individuals by sequencing genomic (g)DNA in the forward (F) and reverse (R) directions but allelic origin could not be assigned without parental samples. The SNP is indicated by a vertical line. **(D,E)** Human fetal heart allele-specific assays, informative samples from 15 individual hearts with both fetal and maternal DNA samples. SNPs annotated in the UCSC genome browser were identified by sequencing with flanking primers in gDNA from the mother (single peak) and fetus (double peak) in the upper panels. Allele-specific assays amplifying and sequencing cDNA from fetal heart RNA are in the lower panels. Sample 11,886 has both peaks indicating expression from the maternal and paternal alleles whereas mono-allelic paternal expression of DDC_EXON1A is detected in 11,908.

The organization of the *Ddc/Grb10* locus is conserved between mouse and human where it is located on Chromosome 7 in the human genome (Hitchins et al., 2002). Studies have shown that *DDC* is expressed from both parental alleles in several tissues from six human fetuses (Hitchins et al., 2002) but heart had not been assayed. We sequenced for SNPs in 25 human fetal hearts to test for monoallelic and parent-of-origin-specific expression of *DDC_EXON1A*. A SNP was present in three informative samples, two displayed mono-allelic expression (Figures 4A,B), the third showed a biased expression (Figure 4C). A further 15 fetal heart samples were collected with matched maternal genomic DNA and these were sequenced for SNPs. A SNP was found in two informative samples, sample 11,886 showed biased expression from the paternal allele (Figure 4D) and 11,908 showed paternal expression (Figure 4E). Polymorphic imprinting patterns are consistent with findings at other human imprinted loci such as *IGF2* (Giannoukakis et al., 1996) where inter-individual variation in parental allele-specific expression/imprinting has been documented as well as more broadly at other imprinted loci across the genome (Zink et al., 2018).

## Discussion

### *Ddc_exon1a* Imprinted Expression

*Ddc_exon1a* is paternally expressed in the developing mouse heart. Immunostaining of sectioned mouse hearts reveals strong signal in the region of the secondary heart field where progenitor cells go on to form the distal parts of the outflow tract and arterial trunks, right ventricle and interventricular system. Abnormal development in any of these components of cardiac development can result in congenital heart disease (Chaudhry et al., 2014; Kelly et al., 2014) and the importance of right ventricular abnormalities in the pathology of cardiovascular disease in the adult has recently been an intense are of investigation (Amsallem et al., 2018).

As is typical for imprinted genes, *Ddc_exon1a* exists in an imprinting cluster, sharing an imprinting control region (ICR) with the *Grb10* gene. The deletion of the ICR on the paternal allele in mouse heart results in the silencing of the active paternal *Ddc_exon1a* allele indicating that imprinted *Ddc_exon1a* expression in heart is governed by the ICR via a *cis*-acting mechanism. Deletion of the maternally inherited ICR does not alter expression of *Ddc_exon1a* in heart (Shiura et al., 2009) because the maternal allele is normally epigenetically silenced. Investigating the tissue distribution of *Ddc_exon1a* and *Grb10* is an important step for examining the regulatory relationship between these two clustered genes. Given that *Ddc_exon1a* is highly tissue-specific in its expression, the spatial distribution of these genes was examined in the developing heart and appeared to be non-overlapping. Paternal *Grb10* gene expression and DDC protein localization (Figure 2) suggests that paternal *Ddc_exon1a* and paternal *Grb10* imprinted gene expression is not coupled in the heart.

*Grb10* is also oppositely imprinted (paternally expressed) in the brain compared to most other tissues and this suggested that brain could also be a useful tissue in which to examine the imprinted expression of *Ddc_exon1a* for the identification of tissue-specific imprinting mechanisms. However, *Ddc_exon1a* is not imprinted in the brain of neonatal mice (Supplementary Figure 2) implying that the epigenetic control of *Ddc_exon1a* imprinting is unlikely to be co-ordinated with *Grb10* in brain, although paternal expression was reported in NSCs. The ICR seems only to influence tissue-specific imprinting of *Ddc_exon1a* in the heart. It is possible that at the individual cell type level, *Ddc_exon1a* could be imprinted in brain because *Grb10* exhibits imprinting in neurons, but the signal is masked by the maternal expression of *Grb10* in glia (Yamasaki-Ishizaki et al., 2007) and so there could be some cell-specific expression co-ordination (Tucci et al., 2019).

Antisense transcripts are involved in imprinted gene regulation at several well characterized loci (Sleutels et al., 2002). If the *AK006690* transcript was involved in the mechanism of imprinting at *Ddc_exon1a*, it would be predicted to be maternally expressed in heart based on imprinting mechanisms at other loci. However, we detected biallelic (at E15.5) or paternally biased expression (E16.5 and nb) which discounts an obvious mechanistic role for *AK006690* in the imprinting of *Ddc_exon1a*.

### DDC Function in Heart

Homozygous null mice for *Ddc* die late in prenatal development (Eppig et al., 2012), likely due to a lack of neurotransmitter synthesis in the brain and CNS. However, mice harboring a deletion of the *Ddc_exon1a* allele inherited through the paternal line only (*Ddc^*Pat*Δ^*, are knockouts for *Ddc* in heart due to its imprinted status and the expression pattern of the *Ddc_Exon1A* isoform. In the small number of animals studied, compared to the wildtype littermates, *Ddc^*PAT*Δ^* mice tend to have right ventricular hypoplasia of the myocardium at the region that exhibits the most abundant *Ddc* expression at E15.5 (Figure 3 and Supplementary Figure 3) pointing to a role for DDC in myocardial growth. *Ddc* is expressed mainly in the myocardium of the right ventricle and IVS. These structures are derived from the secondary heart field (SHF) population of myocardial progenitors (Kelly, 2012) further delineating the SHF as a unique myocardial population distinct from the first heart field (FHF) and suggesting that *Ddc* plays a role in SHF ontogeny.

Gene expression differences between *Ddc^*PAT*Δ^* and Ddc^*WT*^ hearts were found at *Ddc* itself, with only modest differences of other genes (Supplementary Table 1). DDC may not therefore function to directly mediate gene expression in the heart, but instead results in biochemical changes that influence local gene expression via feedback mechanisms. Of note, *Ddc* expression is not ubiquitous throughout the heart and is not expressed in all ventricular myocytes. There is also no detectable expression in other cardiac cell populations such as the endocardium or epicardium. This heterogeneity of expression, with expression limited to the septum and apical portion of the RV may mask changes in gene expression in *Ddc^*PAT*Δ^* cells when pooled in bulk cell analyses such as microarrays with cell populations not affected by alterations in *Ddc* expression. Further evaluation of the impact of DDC deletion will require single cell transcriptomic analysis.

### Human *DDC_EXON1A* Imprinting

*DDC_EXON1A* displays polymorphic monoallelic expression in the developing human heart, and where there is monoallelic expression, this is from the paternal allele as observed in the mouse. The NHGRI-EBI Catalog of published genome-wide association studies does not currently report mutations in DDC that relate to heart development or cardiomyopathy. However, hypermethylation of the GRB10 ICR in peripheral blood samples has recently been associated with congenital heart disease (Chang et al., 2021). The complex pattern of tissue-specific imprinted expression at this locus suggests it may warrant special consideration in genetic studies because *Ddc_exon1a* ablation has a mild effect on the developing heart and with a small effect size there could be moderately widespread ablation of this exon in human populations that presents a suitable genetic background for other mutations to cause developmental abnormalities.

In summary, *Ddc_exon1a* is a heart-specific imprinted isoform expressed from the paternally inherited allele regulated by differential DNA methylation at an ICR in the adjacent *Grb10* gene but not by the expression of *Grb10* itself. When ablated via gene knock-out in the heart, morphological changes are detected in small numbers of embryos. It is important to note that RV function was not assessed and it is reasonable to suspect that abnormal RV function might contribute to the late embryonic lethality observed in *Ddc* mutants. In humans, *DDC_EXON1A* gene expression has a paternal bias and is polymorphically imprinted, a finding common among imprinted genes in humans (Monk et al., 2006).

## Data Availability Statement

The datasets presented in this study can be found in online repositories. The microaray data have been deposited in GEO, accession number GSE87595.

## Ethics Statement

The studies involving human participants were reviewed and approved by the Joint Research Ethics Committee of London, Camberwell St Giles, project ID 53717. The patients/participants provided their written informed consent to participate in this study. The animal study was reviewed and approved by the *Ddc* knockout model generation [DdcGt(OST129277)Lex] [B6;129S5-DdcGt(neo)420Lex, ID# 11693–UCD] was carried out by Lexicon Genetics Inc., United States and breeding, genotyping and tissue acquisition by the UC Davis mouse biology program. Only frozen tissues were shipped out to the laboratory. Work involving *Grb10* animals was approved by the University of Bath Animal Welfare and Ethical Review Body and carried out under UK Home Office license (PPL 30/2839).

## Author Contributions

AP conducted experiments, interpreted data and contributed to writing the manuscript. SA, MF, MC, WP, MS, and BM conducted the experiments. MI collected human tissue samples. BM, NB, and RS designed and performed data analyses. MM-S and AW provided and collected *Grb10* transgenic tissue samples. HB conceived the EFIC analysis and provided EFIC facilities, supervised AP, and interpreted the heart sections and EFIC data. RO conceived and supervised the project, interpreted the data, and wrote the manuscript with AP. All authors contributed to the critical reading and editing of the manuscript.

## Conflict of Interest

The authors declare that the research was conducted in the absence of any commercial or financial relationships that could be construed as a potential conflict of interest.

## Footnotes

1 http://www.informatics.jax.org/external/ko/lexicon/2095.html

2 http://www.hdbr.org
