# Supplementary material for: Imprinted Gene Expression and Function of the Dopa Decarboxylase Gene in the Developing Heart
Source: Front Cell Dev Biol. 2021 Jun 22;9:676543. doi: 10.3389/fcell.2021.676543 (PMC8258389; doi:10.3389/fcell.2021.676543)
Supplement: Supplementary file 1 [file Image_1.pdf]

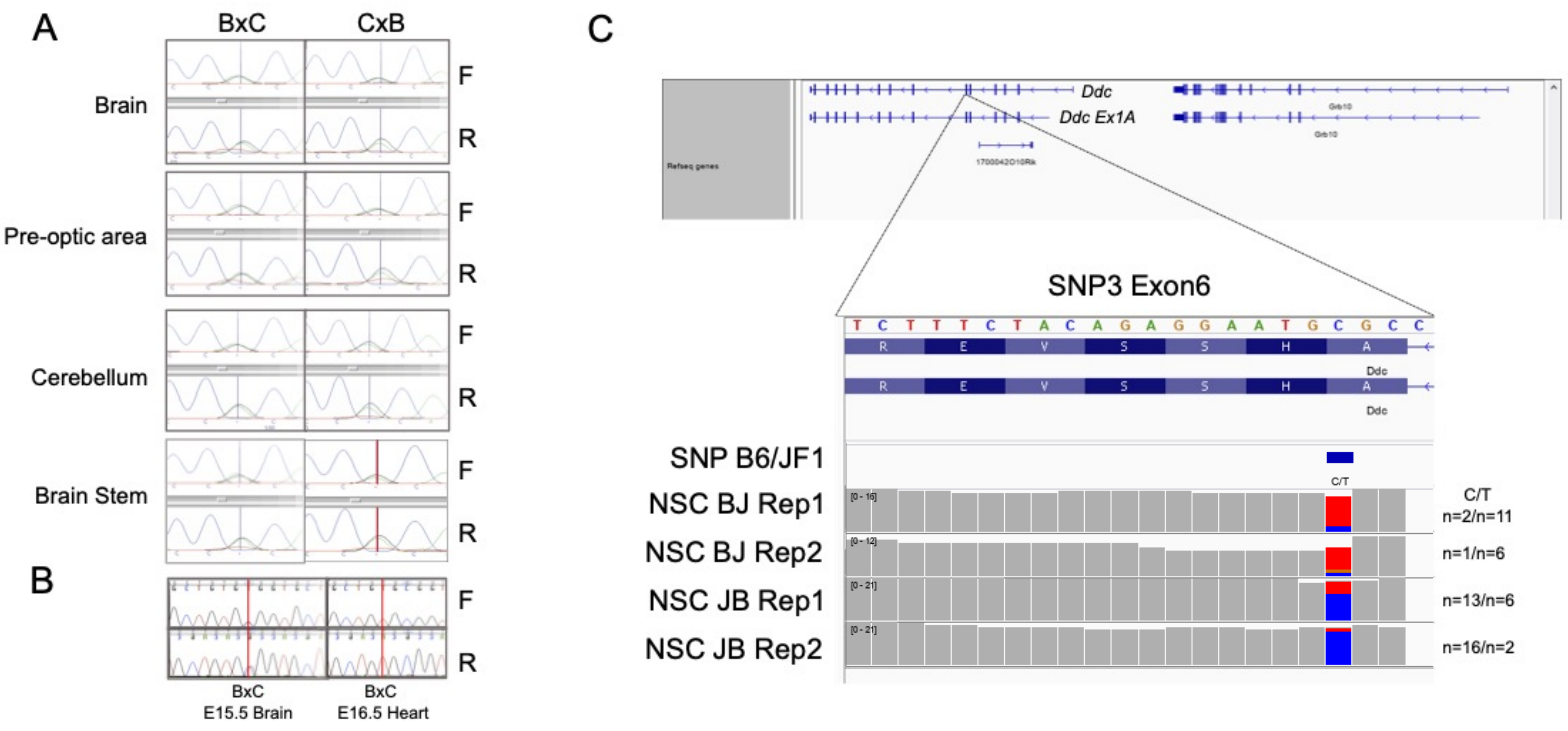

D

	SNP1 (Ex2)			SNP2 (Ex3)			SNP3 (Ex6)			SNP4 (Ex8)			SNP5 (Ex15)			SNP6 (Ex15)		
	C (Bl6)	T (JF1)	Allelism	A (Bl6)	G (JF1)	Allelism	C (Bl6)	T (JF1)	Allelism	C (Bl6)	T (JF1)	Allelism	T (Bl6)	C (JF1)	Allelism	T (Bl6)	C (JF1)	Allelism
NSC BJ Rep1	0	8	Pat (100%)	0	7	Pat (100%)	2	11	Pat (85%)	1	7	Pat (87%)	0	10	Pat (100%)	0	5	Pat (100%)
NSC BJ Rep2	0	2	Pat (100%)	0	4	Pat (100%)	1	6	Pat (86%)	2	11	Pat (78%)	0	5	Pat (100%)	0	3	Pat (100%)
NSC JB Rep1	4	8	Mat (67%)	14	6	Pat (70%)	13	6	Pat (81%)	5	3	Pat (62%)	5	2	Pat (71%)	2	1	Pat (67%)
NSC JB Rep2	5	0	Pat (100%)	4	1	Pat (80%)	16	2	Pat (89%)	8	0	Pat (100%)	13	7	Pat (65%)	1	1	

Supplementary Figure 1

Allele-specific assays of brain regions, brain and heart to detect parent-of-origin-specific gene expression of Ddc_exon1a. RNA from the brain regions indicated (A), from six-week-old mice from reciprocal crosses (BxC indicates the maternal allele is from C57Bl6 and the paternal from Mus mus castaneus, and vice versa for CxB). RT-PCR and Sanger sequencing over regions with known SNPs between strains was used to confirm the imprinting status in the embryonic stage in brain (B) shows BxC E15.5 brain and BxC E16.5 heart. The red vertical bars indicate the SNPs between strains and in brain regions from neonates and E15.5 brain, both alleles are present in roughly equal proportions indicating biallelic expression compared to E16.5 heart where only the single paternally expressed, (imprinted) allele is present. (C) Screenshot from the IGV genome browser (mouse mm9 genome) with the different isoforms found at the locus. A zoom in on one of the SNPs found between C57Bl6 and JF1 strain in Ddc shows the number of reads carrying one allele or the other (T in red, C in blue or grey). Two replicates from reciprocal crosses in neural stem cells (NSC) are shown. (D) Summary tables for the six different SNP localised inside Ddc transcripts. The allele with the highest number of reads shows a bias toward the paternal allele in neural stem cells.
