# Supplementary material for: Imprinted Gene Expression and Function of the Dopa Decarboxylase Gene in the Developing Heart
Source: Front Cell Dev Biol. 2021 Jun 22;9:676543. doi: 10.3389/fcell.2021.676543 (PMC8258389; doi:10.3389/fcell.2021.676543)
Supplement: Supplementary file 2 [file Image_2.pdf]

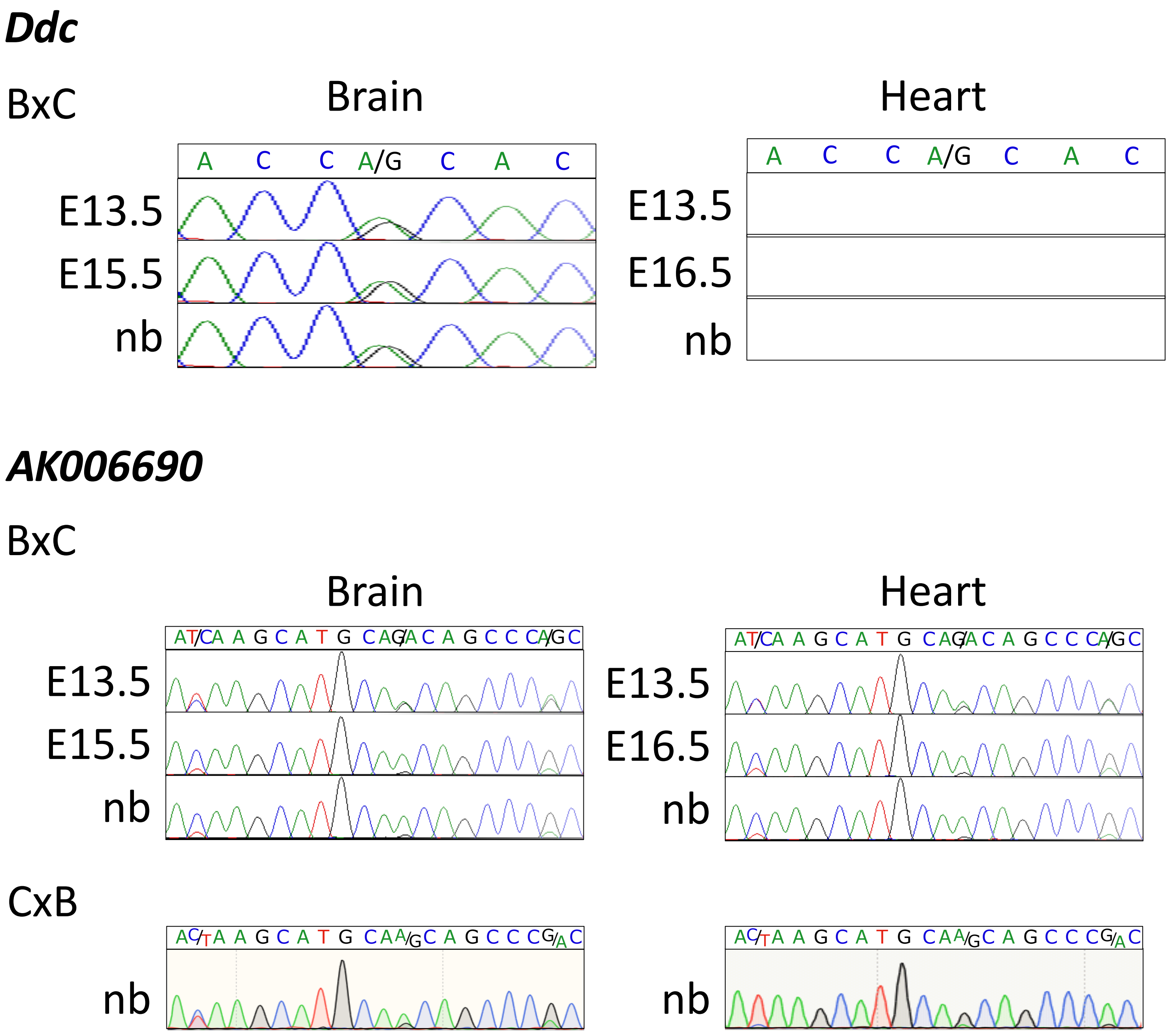

Supplementary Figure2
Allele specific assays of Ddc in e13.5, e15.5 and newborn (nb) BxC brain show biallelic expression (2 peaks at the SNP) whereas in heart at e13.5, e16.5 and nb, paternal expression is revealed by a single peak at the SNP. The AK006690 antisense transcript assay illustrates three inter-sub specific SNPs indicated by the backslash between 2 bases). In brain and heart, the transcript is biallelic at E13.5. At the later stages of E15.5 and newborn brain and E16.5 and newborn heart, a paternal bias in expression is detected. In the reciprocal CxB assay in brain, two of the SNPs are biallelic and one has a slight bias suggesting that there is no strong imprinting in brain. In the CxB reciprocal nb heart, there is a swap over of the parental origin of the expressed allele compared to BxC supporting a parental-specific expression bias in this tissue. B= C57Bl6 strain, C= Mus castaneus strain.
