# Supplementary material for: Imprinted Gene Expression and Function of the Dopa Decarboxylase Gene in the Developing Heart
Source: Front Cell Dev Biol. 2021 Jun 22;9:676543. doi: 10.3389/fcell.2021.676543 (PMC8258389; doi:10.3389/fcell.2021.676543)
Supplement: Supplementary file 3 [file Image_3.pdf]

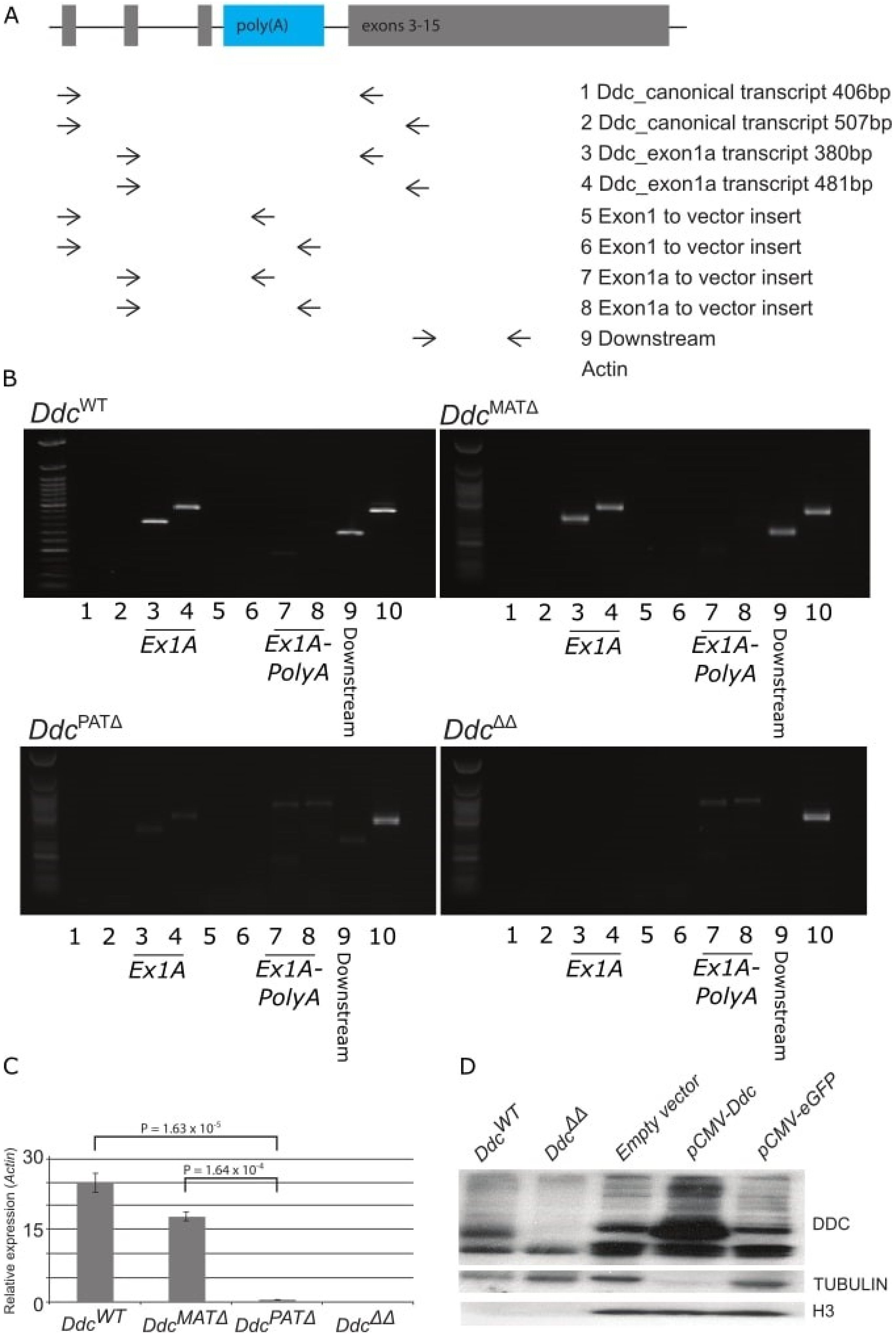
A
poly(A)
exons 3-15
1 Ddc_canonical transcript 406bp
2 Ddc_canonical transcript 507bp
3 Ddc_exon1a transcript 380bp
4 Ddc_exon1a transcript 481bp
5 Exon1 to vector insert
6 Exon1 to vector insert
7 Exon1a to vector insert
8 Exon1a to vector insert
9 Downstream
Actin
B
Ddc^WT
Ddc^MATΔ
Ddc^PATΔ
Ddc^ΔΔ
1 2 3 4 5 6 7 8 9 10
Ex1A
Ex1A-PolyA
Downstream
C
P = 1.63 x 10^-5
P = 1.64 x 10^-4
Relative expression (Actin)
30
15
0
Ddc^WT
Ddc^MATΔ
Ddc^PATΔ
Ddc^ΔΔ
D
Ddc^WT
Ddc^ΔΔ
Empty vector
pCMV-Ddc
pCMV-eGFP
DDC
TUBULIN
H3

Supplementary Figure 3

RNA transcript analysis in Ddc knockout mouse heart at e15.5. (A) primer locations are indicated by black arrows, forward primers were designed in exon 1, exon 1a and exon 4. Reverse primers were designed in exon 3, exon 4 and exon 5, plus two pairs in the VICTR48 vector. RT-PCR experiments (lanes 1-10) are indicated to the right of the primer locations. Results are shown in (B) RT-PCR of Ddc^{WT}, $Ddc^{MAT\Delta}$, $Ddc^{PAT\Delta}$ and $Ddc^{\Delta\Delta}$ expression are as expected except in the $Ddc^{PAT\Delta}$ genptype where Ddc_exon1a expression is present despite the fact that the maternal allele is epigenetically silenced (lanes 3,4). (C) depicts the results of a quantitative PCR analysis in e15.5 hearts for Ddc transcript in Ddc^{WT}, $Ddc^{MAT\Delta}$, $Ddc^{PAT\Delta}$ and $Ddc^{\Delta\Delta}$ genotypes. $Ddc^{PAT\Delta}$ has a diminished expression of Ddc_exon1a as expected. (D) western blot analysis on protein extract from either Ddc^{WT}, or $Ddc^{\Delta\Delta}$ whole carcass and NIH3T3 fibroblast cell lines transfected with either Empty Vector, Ddc under the control of the cauliflower mosaic virus promoter (pCMV Ddc) or eGFP under the control of the cauliflower mosaic virus promoter (pCMV eGFP). The western blot was probed using antibodies raised against DDC, followed by Tubulin and Histone H3 as loading controls. Tubulin detection in the pCMV transfected fibroblast lane is light, possibly because of the high levels of DDC staining at the same location, the blot was re-probed for histone H3 which stained as expected. The RT-PCR (B) was performed once, qPCR for Ddc expression (C) was repeated 4x (Ddc^{WT}), 3x ($Ddc^{MAT\Delta}$), 3x ($Ddc^{PAT\Delta}$) and 2x ($Ddc^{\Delta\Delta}$) and the western blot (D) was performed once.
