# Supplementary material for: Imprinted Gene Expression and Function of the Dopa Decarboxylase Gene in the Developing Heart
Source: Front Cell Dev Biol. 2021 Jun 22;9:676543. doi: 10.3389/fcell.2021.676543 (PMC8258389; doi:10.3389/fcell.2021.676543)
Supplement: Supplementary file 4 [file Table_1.pdf]

s	ID	logFC	t	P.Value	adj.P.Val	B	Symbol							
14908	ILMN_2628647	3.392436935	36.91756326	3.60E-012	1.63E-007	9.435210895	Ddc	increased heart weight	Due to hypertension caused by Cre mediated knockout of Ddc in Kidney	J:175630				
14910	ILMN_1229396	3.340576765	12.4205174	1.79E-007	0.0027029	6.087224847	Ddc	increased heart weight		J:175630				
14909	ILMN_1260450	3.170024081	30.17264868	2.75E-011	6.23E-007	9.136623394	Ddc	increased heart weight		J:175630				
37514	ILMN_2588249	-1.641246041	-4.91626835	0.000578819	0.1432212	0.063057856	S3-12							
40327	ILMN_2988931	-1.620561414	-3.751341251	0.00366632	0.2274714	-1.602336792	Stfa1							
40326	ILMN_2774410	-1.585791832	-3.577257595	0.004902838	0.2505704	-1.867969881	Stfa1							
11295	ILMN_1219583	-1.555826976	-3.616332563	0.004591913	0.2486875	-1.808048234	Capn2	Thin ventricular wall		J:175868				
20494	ILMN_3141257	1.537362603	3.272914852	0.008207916	0.2885579	-2.339620778	Hbb-y							
7639	ILMN_2675874	-1.522587567	-3.597520293	0.00473897	0.2494371	-1.836876464	Alas2							
33883	ILMN_3116570	-1.456427862	-3.955253516	0.00261998	0.2178485	-1.295942715	Pdhb							
37506	ILMN_2803674	-1.381980976	-3.198051902	0.009328101	0.2992686	-2.456717549	S100a9							
12550	ILMN_2817864	-1.274939367	-3.971784993	0.002550134	0.215434	-1.271347887	Ckmt2	dilated heart left ventricle heart left ventricle hypertrophy		J:101961				
11093	ILMN_1222071	-1.27089169	-3.57441803	0.004926267	0.2508813	-1.87233071	C920004C08Rik							
23834	ILMN_2707808	-1.24456192	-3.368763754	0.006972204	0.2763024	-2.19024029	LOC100044756							
11296	ILMN_2683586	-1.204095557	-3.669488117	0.004201448	0.2430087	-1.726803515	Capn2	Thin ventricular wall		J:175868				
15013	ILMN_2705097	1.191770138	4.260032462	0.001601021	0.1982966	-0.848917767	Deadc1							
4079	ILMN_2472708	-1.112398782	-5.79197187	0.000163814	0.1020503	1.164055944	5730402C02Rik							
16621	ILMN_2995688	-1.110740168	-3.61054168	0.00463667	0.2486875	-1.816918183	EG433016							
33882	ILMN_1247561	-1.086886008	-4.471764206	0.001145437	0.1848813	-0.546831601	Pdhb							
42047	ILMN_2460136	1.075977427	3.771064346	0.003548316	0.2255842	-1.572466712	Tnnt1							
18303	ILMN_2482572	-1.067644828	-3.202024409	0.009264898	0.2989456	-2.450495858	Flnb							
42958	ILMN_2502542	-1.06257754	-3.176824319	0.009673386	0.3027095	-2.489977923	Uap1							
12551	ILMN_2698052	-1.052170087	-3.349310105	0.007206529	0.2783454	-2.220504006	Ckmt2	dilated heart left ventricle heart left ventricle hypertrophy		J:101961				
36499	ILMN_2733708	-1.010553354	-3.325270758	0.007507325	0.2821829	-2.257941485	Rbp7							
14931	ILMN_2693185	-1.009306607	-4.506182034	0.00108539	0.1836985	-0.498415669	Ddo							
38982	ILMN_2696610	-1.005918735	-3.408311278	0.006519722	0.2705954	-2.128810373	Slc25a37							
29947	ILMN_2696609	-0.992410044	-3.687006534	0.004080453	0.2387171	-1.700098214	Mscp							
43021	ILMN_2486267	-0.963088847	-3.376608513	0.006879944	0.2750624	-2.178044755	Ube2l6							
9967	ILMN_2700239	-0.955654701	-3.973251884	0.002544031	0.215434	-1.269167357	BC100530							
5825	ILMN_1226919	-0.942494706	-3.497571152	0.005606382	0.2595732	-1.99066008	A330102I10Rik							
22490	ILMN_2974117	-0.920180282	-3.25572132	0.008452352	0.2919985	-2.366484073	Kctd6							
30299	ILMN_2640008	-0.900709002	-4.086154648	0.002117323	0.2102511	-1.10226006	Myh6	dilated cardiomyopathy dilated heart left ventricle abnormal heart left ventricle morphology cardiac fibrosis cardiac hypertrophy disorganized myocardium abnormal heart atrium morphology abnormal heart morphology abnormal myocardial fiber morphology cardiac interstitial fibrosis disorganized myocardium		J:114549	J:104363	J:32960	J:51939	Many more
15147	ILMN_1219915	-0.900650458	-3.818974929	0.003277856	0.2245934	-1.500110592	Dgat2							
33881	ILMN_3041839	-0.884885562	-3.379512974	0.006846105	0.2750624	-2.173530703	Pdhb							
18301	ILMN_2482571	-0.882425455	-3.459495947	0.005978651	0.263656	-2.049501888	Flnb							
12963	ILMN_1246054	0.873094368	6.397181721	7.29E-005	0.0988892	1.847712873	Col2a1	abnormal heart valve morphology		J:30041				
5276	ILMN_1225181	-0.862032248	-6.555349653	5.94E-005	0.0988892	2.016380642	9626962_211_rc							
29324	ILMN_2700408	-0.842202612	-4.370967696	0.00134236	0.1909407	-0.68974127	Mgll							
22088	ILMN_1213858	-0.830642918	-3.385004281	0.006782596	0.2743273	-2.164998094	Itgb3bp							
34348	ILMN_2479717	0.827424871	6.353141086	7.72E-005	0.0988892	1.800024792	Pilra							
6904	ILMN_1227579	-0.816141847	-6.565003503	5.87E-005	0.0988892	2.026544702	Acot11							
4414	ILMN_2594139	0.814373223	4.233748249	0.001669687	0.1989607	-0.886916658	6330403K07Rik							
43894	ILMN_1234111	-0.812593971	-3.834117238	0.003196919	0.2231222	-1.477303174	Vtn							
1675	ILMN_3158565	-0.811785676	-3.930888659	0.002726594	0.2182072	-1.33226095	2310076L09Rik							
14929	ILMN_2808191	-0.807585042	-5.477008481	0.000254732	0.1143099	0.783510149	Ddo							
11835	ILMN_2688416	-0.80558831	-4.316216913	0.001464022	0.1946082	-0.76805629	Cd300lg							
3703	ILMN_1213433	-0.805118514	-4.278149547	0.001555426	0.197046	-0.822788993	4933406L09Rik							
13009	ILMN_2945030	0.795550376	7.327644491	2.30E-005	0.0941113	2.783750212	Col9a2							
35595	ILMN_1239724	0.791333604	3.367483093	0.006987386	0.2763024	-2.192231683	Psmb1							
37634	ILMN_3008068	-0.790501623	-4.253610052	0.00161752	0.1982966	-0.858192637	Scara5							
6953	ILMN_2622671	-0.787012126	-3.931181227	0.002725287	0.2182072	-1.331824363	Acsl1	cardiac hypertrophy cardiac hypertrophy increased heart weight enlarged heart heart left ventricle hypertrophy thick ventricular wall enlarged heart heart left ventricle hypertrophy thick ventricular wall abnormal myocardial fiber morphology cardiac hypertrophy cardiac interstitial fibrosis enlarged heart		J:68639				
38169	ILMN_2604224	0.781513058	3.601584172	0.004706794	0.2489817	-1.830645664	Sema5a							
7233	ILMN_2675064	-0.779666249	-3.16591331	0.009855921	0.3052571	-2.507082621	Adhfe1							
28565	ILMN_2483304	-0.772915889	-4.242213333	0.00164724	0.1989607	-0.874666986	Lyve1							
39739	ILMN_2616196	0.763456075	3.710679988	0.003922676	0.2350511	-1.664067098	Sox11	Many mophological defects	J:175338					
38276	ILMN_1220234	-0.735648971	-3.588585214	0.004810518	0.2495827	-1.850582093	Serpina1e							
44776	ILMN_1257178	0.735325644	6.555734062	5.94E-005	0.0988892	2.016785652	Zfp367							
29130	ILMN_2860964	-0.734528305	-5.337600699	0.000311088	0.117837	0.60954788	Med23							
38490	ILMN_2657543	-0.721372122	-4.046007862	0.002259744	0.2114221	-1.16139918	Sgcg	abnormal heart ventricle morphology decreased heart weight thick ventricular wall	J:49871					
16926	ILMN_2605819	-0.70683846	-3.327666535	0.007476776	0.2821829	-2.254208515	Egln3							
12526	ILMN_2718330	-0.706633577	-3.180276257	0.00961636	0.302271	-2.484567695	Cish							

44163	ILMN_2511913	0.704820901	5.903417873	0.000140586	0.1020503	1.294599155	Wnt5a						
7861	ILMN_2844820	0.702654586	3.866701364	0.003029746	0.2231222	-1.428326208	Angptl7						
12964	ILMN_2759142	0.698928169	6.364646663	7.60E-005	0.0988892	1.812513898	Col2a1	abnormal heart valve morphology		J:30041			
25397	ILMN_2777427	-0.692868577	-4.819491468	0.000669948	0.1519947	-0.066846509	LOC329416						
41452	ILMN_2665133	-0.68767619	-3.765681371	0.003580125	0.225973	-1.5806143	Timp4	Abnormal heart right ventricle morphology increased left ventricle weight	J:165901				
19020	ILMN_1249941	-0.683456029	-4.053765117	0.002231461	0.2114221	-1.149953821	Gdf10						
41378	ILMN_2736451	0.679600737	6.157624556	9.98E-005	0.0988892	1.584454549	Thsd2						
29072	ILMN_3158499	0.675752188	4.568423358	0.000985096	0.1789736	-0.411359924	Mdk						
14282	ILMN_1236811	-0.671597739	-3.790691848	0.003434807	0.2245934	-1.542789502	D330008I21Rik						
35499	ILMN_2734036	0.671455927	4.601749526	0.00093547	0.1785243	-0.365013791	Prrx2						
17156	ILMN_1247358	0.666576926	5.565513645	0.000224697	0.1138323	0.892187532	Emid2						
31289	ILMN_3127391	0.665563717	3.157806275	0.009993823	0.3069005	-2.519795359	Npr3						
31190	ILMN_2821676	-0.66433233	-4.627833156	0.000898478	0.1771726	-0.328870239	Nostrin						
38274	ILMN_3004600	-0.663862862	-3.475242948	0.005821685	0.2622526	-2.025149873	Serpina1c						
29071	ILMN_1254450	0.657897138	3.881831998	0.002955247	0.2222866	-1.405631145	Mdk						
29680	ILMN_2977492	0.657439346	5.219462621	0.000369301	0.1185981	0.45945799	Moxd1						
33518	ILMN_1237631	0.653114415	3.648741793	0.004349573	0.2454503	-1.758475076	Pard6g						
1603	ILMN_2493030	-0.652013054	-5.127754657	0.000422482	0.1266915	0.341256865	2310043N10Rik						
19816	ILMN_2754155	0.650273939	3.712345351	0.003911818	0.2349218	-1.661534902	Gpr126	dilated heart ventricle thin myocardium		J:167309			
13051	ILMN_2549929	-0.649551079	-6.544810058	6.02E-005	0.0988892	2.005266958	Copg2as2						
22668	ILMN_1254031	-0.645758077	-4.460502806	0.001165839	0.1848813	-0.562715591	Klf9						
17285	ILMN_2720813	-0.642761648	-3.375501855	0.006892883	0.2750624	-2.179764879	Epb4.1	abnormal heart development		J:122548			
13231	ILMN_2645816	0.6375069	5.284974651	0.000335709	0.117837	0.542991221	Cpxm1						
15105	ILMN_2697361	0.636179912	4.132276447	0.001965257	0.2067957	-1.034613177	Dennd2a						
29254	ILMN_2633350	0.635436933	4.597067934	0.000942278	0.1785243	-0.371513125	Mfap4						
10430	ILMN_2463583	0.626904846	6.675759168	5.10E-005	0.0988892	2.14208986	C030009J22Rik						
21453	ILMN_1219335	0.625734076	3.444381494	0.006133424	0.2657438	-2.072896988	Igfbp3						
27418	ILMN_1243991	0.623552489	3.627055467	0.004510218	0.2478204	-1.791633572	LOC433476						
21272	ILMN_2667814	-0.623483377	-4.518814827	0.001064193	0.1832233	-0.480694215	Iars2						
25069	ILMN_1252353	0.623352466	3.611946234	0.004625773	0.2486875	-1.814766477	LOC241621						
38817	ILMN_2827072	0.620274835	5.830640338	0.000155319	0.1020503	1.209592113	Slc13a4						
33467	ILMN_2946088	0.614307481	5.594449838	0.000215718	0.1138323	0.927422376	Panx1						
19370	ILMN_1238310	0.61306645	4.305218225	0.001489835	0.1946082	-0.78384646	Gm1673						
37236	ILMN_2590860	0.61109959	3.907203912	0.002834587	0.2210895	-1.367643616	Rpo2tc1						
18315	ILMN_1229680	0.607853521	7.272087807	2.45E-005	0.0941113	2.731546562	Flrt3	abnormal heart development cardia bifida		J:136719	J:142039		
29323	ILMN_2857957	-0.601807711	-3.308989341	0.007718354	0.2860023	-2.283321309	Mgll						
659	ILMN_1225662	-0.601444157	-3.571023057	0.004954431	0.251504	-1.877545609	1700016J18Rik						
23334	ILMN_1247916	-0.601012095	-3.438218966	0.006197711	0.2670205	-2.082441619	Lims2	abnormal heart atrium auricular region morphology abnormal heart left ventricle morphology abnormal heart shape abnormal heart ventricle morphology abnormal myocardium compact layer morphology abnormal ventricle myocardium morphology cardiac fibrosis dilated heart atrium dilated heart left ventricle small myocardial fiber		J:167114			
43289	ILMN_2936646	0.59899439	3.607006011	0.004664218	0.2486875	-1.822335595	Upk1b						
41047	ILMN_2930707	0.596100782	5.398367204	0.000285037	0.117837	0.685795196	Tcf21	hemopericardium	J:55785				
34408	ILMN_1220244	0.595723226	3.446882575	0.00610753	0.2657438	-2.069024236	Pitx2	abnormal atrioventricular cushion morphology atrial septal defect atrioventricular septal defect common atrioventricular valve dextrocardia double outlet heart right ventricle failure of atrioventricular cushion closure right atrial isomerism transposition of great arteries ventricular septal defect and many more		J:128475	J:69854	J:57674	J:55455
4416	ILMN_1245549	0.594864267	4.305089672	0.001490139	0.1946082	-0.78403113	6330404C01Rik						
11750	ILMN_1255422	-0.594357709	-4.2771594	0.001557882	0.197046	-0.824215666	Ccrn4l						
3120	ILMN_2752300	-0.592250112	-3.260573577	0.008382622	0.2913074	-2.358900965	4930452B06Rik						
15349	ILMN_1237061	-0.591362197	-3.292739926	0.007935051	0.286779	-2.308669919	Dm15	abnormal myocardial fiber morphology cardiac interstitial fibrosis heart left ventricle hypertrophy thick interventricular septum thick ventricular wall		J:33711	J:93614		
20512	ILMN_2507044	-0.587953865	-4.389104099	0.001304449	0.1909008	-0.66390542	Hcfc2						
37540	ILMN_1252794	0.587890213	5.086748338	0.000448852	0.12825	0.287926911	Sall3						
44492	ILMN_2441346	-0.587550162	-3.605406248	0.004676739	0.2486875	-1.824787226	Zcchc5						
17918	ILMN_2449644	0.586798411	4.347443382	0.001393273	0.1935239	-0.723331365	Fbln1	abnormal heart morphology atrial septal defect double outlet heart right ventricle muscular ventricular septal defect ostium primum atrial septal defect ostium secundum atrial septal defect overriding aorta perimembraneous ventricular septal defect ventricle myocardium hypoplasia ventricular septal defect		J:137690			
32694	ILMN_1243468	-0.585809339	-3.539594925	0.005223208	0.2548622	-1.925877907	Olfr608						
34404	ILMN_3118071	0.581716182	4.102978034	0.002060472	0.2083915	-1.077548508	Pitx2	abnormal atrioventricular cushion morphology atrial septal defect atrioventricular septal defect common atrioventricular valve dextrocardia double outlet heart right ventricle failure of atrioventricular cushion closure right atrial isomerism transposition of great arteries ventricular septal defect and many more		J:128475	J:69854	J:57674	J:55455
17444	ILMN_1257143	-0.579687459	-3.514819853	0.005445706	0.2570402	-1.964049155	Ermap						

21462	ILMN_2764588	-0.575727898	-3.563058797	0.005021158	0.251808	-1.889783955	Igfbp7		
29074	ILMN_3079236	0.575327265	4.751253821	0.000743299	0.161814	-0.159415651	Mdk		
43305	ILMN_1252263	-0.574421534	-3.850704869	0.003110645	0.2231222	-1.452353008	Uqcrb		
12934	ILMN_1222111	-0.571297094	-3.847875666	0.003125186	0.2231222	-1.456605988	Col15a1		
19959	ILMN_1243154	-0.569508465	-5.094969035	0.000443428	0.12825	0.298641804	Gpsm1		
30317	ILMN_2768252	0.565906852	3.807471414	0.00334077	0.2245934	-1.517456979	Myl7	abnormal atrioventricular cushion morphology abnormal heart looping abnormal heart tube morphology abnormal myocardial fiber morphology dilated heart left ventricle enlarged heart atrium pericardial edema trabecula carnea hypoplasia ventricle myocardium hypoplasia	J:87043
39375	ILMN_1253797	0.562866071	3.811993639	0.003315889	0.2245934	-1.510635799	Slit2		
20434	ILMN_2841280	-0.559966412	-6.968168446	3.54E-005	0.0988892	2.437894399	Habp4		
20947	ILMN_2681195	0.559274922	4.652001909	0.000865582	0.1765515	-0.295482787	Hnt		
24816	ILMN_2529682	0.558104956	3.489040429	0.005687653	0.2608203	-2.00383169	LOC230896		
23333	ILMN_2738345	-0.557026025	-3.168302167	0.009815658	0.3044266	-2.503337223	Lims2	abnormal heart atrium auricular region morphology abnormal heart left ventricle morphology abnormal heart shape abnormal heart ventricle morphology abnormal myocardium compact layer morphology abnormal ventricle myocardium morphology cardiac fibrosis dilated heart atrium dilated heart left ventricle small myocardial fiber	J:167114
40317	ILMN_2777319	0.556478645	4.113433375	0.00202595	0.2083915	-1.062212028	Stc1		
5429	ILMN_1249945	-0.553348562	-4.708739571	0.000793272	0.1674436	-0.217493638	9930004G02Rik		
29021	ILMN_2851040	0.550641223	3.96324402	0.002585973	0.2168435	-1.284050015	Mcm3		
30413	ILMN_2694687	-0.549828192	-6.957470125	3.59E-005	0.0988892	2.427304457	Mypn		
5273	ILMN_1240922	-0.547504963	-7.600496839	1.67E-005	0.0941113	3.033706606	9626953_2_rc		
24033	ILMN_2762925	0.543907319	3.582143567	0.004862794	0.2497766	-1.860468294	LOC100046259		
41631	ILMN_2804559	0.543833217	5.232798774	0.000362183	0.1184373	0.476523785	Tmem108		
20154	ILMN_1228233	-0.539117253	-3.453666415	0.006037863	0.2649229	-2.058522757	Gstm1		
24089	ILMN_1244879	-0.537352536	-4.860735654	0.000629373	0.1481763	-0.011285578	LOC100046690		
25784	ILMN_2526333	-0.536140149	-4.196660925	0.001771893	0.2023173	-0.940715983	LOC381284		
35978	ILMN_2764819	0.534898559	6.921079985	3.75E-005	0.0988892	2.391152113	Rab1		
33816	ILMN_2717749	0.534491766	5.047250724	0.000475915	0.1290412	0.236280426	Pde1a		
29191	ILMN_2709087	-0.533908351	-3.751983144	0.003662416	0.2274714	-1.601363908	Meox2	thin myocardium	J:123197 J:136243 J:111702 J:147154
15637	ILMN_2615096	0.531095886	5.230179321	0.000363569	0.1184373	0.473174232	Dpp4		
15	ILMN_2543688	0.530515367	7.276862282	2.44E-005	0.0941113	2.736050546	0610007N19Rik		
7919	ILMN_2741117	-0.529942976	-5.173810155	0.000394822	0.1220628	0.400801714	Ankrd23		
9701	ILMN_2746830	-0.529873947	-3.310064859	0.007704228	0.2860023	-2.281644184	BC022224		
33494	ILMN_2466809	0.527589064	3.265939824	0.008306192	0.2897632	-2.350516332	Papss2		
6686	ILMN_2686700	-0.525367973	-3.330071019	0.007446245	0.2817076	-2.250462401	Abca8a		
23332	ILMN_2942674	-0.524761713	-3.622456683	0.004545069	0.2479674	-1.798671858	Lims2	abnormal heart atrium auricular region morphology abnormal heart left ventricle morphology abnormal heart shape abnormal heart ventricle morphology abnormal myocardium compact layer morphology abnormal ventricle myocardium morphology cardiac fibrosis dilated heart atrium dilated heart left ventricle small myocardial fiber	J:167114
37034	ILMN_2636349	0.524738322	5.09741806	0.000441826	0.12825	0.301831584	Rnf208		
33821	ILMN_1223293	-0.524071077	-4.052272998	0.002236873	0.2114221	-1.152154673	Pde1c		
44635	ILMN_1257607	0.523938516	3.759109719	0.003619361	0.2269921	-1.590565923	Zfp184		
34951	ILMN_2710139	-0.522978251	-6.875495608	3.97E-005	0.0988892	2.345579103	Ppargc1a	Abnormal heart weight	J:111072 J:96306
8495	ILMN_1223147	-0.518517057	-3.340336436	0.007317336	0.2798448	-2.234474011	Art3		
19152	ILMN_1248457	-0.518341318	-3.366441878	0.006999754	0.2763024	-2.193850839	Gipr		
12965	ILMN_2759144	0.518164149	3.891614446	0.002908102	0.2215772	-1.390974327	Col2a1	abnormal heart valve morphology	J:30041
29247	ILMN_1240458	0.51814492	5.59049062	0.000216924	0.1138323	0.922609956	Mfap2		
36820	ILMN_1237773	0.516658669	4.26282417	0.001593904	0.1982966	-0.844888159	Rhou		
18928	ILMN_1219184	0.515478838	3.357886953	0.007102235	0.2773512	-2.207157519	Gats		
38614	ILMN_1246285	0.51464918	3.927346014	0.002742469	0.2182072	-1.337548432	Shisa2		
13589	ILMN_2904703	-0.514000193	-6.292491118	8.35E-005	0.0988892	1.733830788	Ctsl		
6574	ILMN_2913989	-0.51288697	-4.090761182	0.002101595	0.2096086	-1.095489445	AA407270		
37381	ILMN_3162785	0.510413694	3.832772982	0.003204019	0.2231222	-1.479326698	Rspo3		
24259	ILMN_1217043	-0.508809234	-3.335138209	0.007382324	0.2811013	-2.242569271	LOC100047762		
29249	ILMN_2981542	0.508529292	5.730006726	0.000178483	0.1036142	1.09054821	Mfap2		
13665	ILMN_2737302	0.507590155	4.605835649	0.00092957	0.1785243	-0.35934415	Cxcl12	perimembraneous ventricular septal defect	J:34750
1638	ILMN_2430906	-0.506956695	-4.237842786	0.001658789	0.1989607	-0.880990134	2310051E17Rik		
37493	ILMN_1225602	-0.506884395	-3.626398582	0.004515179	0.2478204	-1.792638771	S100a1		
17265	ILMN_3149143	-0.504618759	-4.652549178	0.000864853	0.1765515	-0.29472792	Entpd5		
37556	ILMN_1254210	0.504524487	3.301394299	0.007818872	0.2861986	-2.295167069	Samd5		
13007	ILMN_2439360	0.502659479	3.411619854	0.006483266	0.2701998	-2.123676982	Col9a1		
36797	ILMN_1252902	0.502168759	3.876553681	0.002981016	0.2228076	-1.413544847	Rhobtb1		
33995	ILMN_2987862	-0.501479864	-5.522393611	0.000238827	0.1138323	0.839410445	Per2		
18409	ILMN_1228748	-0.501025875	-3.754968283	0.003644317	0.2274714	-1.596840148	Foxa3		
40489	ILMN_2745370	-0.499796376	-4.100790766	0.002067772	0.2083915	-1.080758973	Sult1a1		
12118	ILMN_2708203	0.498969836	3.431025018	0.006273637	0.2674921	-2.093587982	Cdkn1c		
6982	ILMN_2738825	0.49878846	3.283857608	0.008056133	0.2868074	-2.322533728	Acta1		
12814	ILMN_2596369	0.497659739	4.103106921	0.002060043	0.2083915	-1.077359351	Cnn2		
34478	ILMN_2974343	-0.494945604	-3.787802786	0.003451275	0.2245934	-1.54715479	Pla1a		

19371	ILMN_2802311	0.494816095	4.182661757	0.001812164	0.2030807	-0.961078417	Gm1673					
38421	ILMN_1231689	0.494409986	4.189734472	0.001791699	0.2030346	-0.950787073	Sfrp1	increased heart weight	J:89547			
2181	ILMN_2714278	0.490575627	6.099133021	0.00010792	0.0988892	1.518726715	2810046M22Rik					
28218	ILMN_2782041	0.490097396	3.267415864	0.008285294	0.2897632	-2.348210376	Lphn3					
20461	ILMN_2753342	0.489800863	3.778761018	0.003503346	0.2245934	-1.560823421	Hapln1					
12657	ILMN_1234165	0.488957598	3.434607925	0.006235703	0.2674921	-2.088036026	Clec4g					
29697	ILMN_2703886	0.488479376	3.586100153	0.004830616	0.2495827	-1.854395474	Mphosph6					
39978	ILMN_2909238	-0.48757901	-3.69727094	0.004011248	0.2368101	-1.684467628	Spnb1	abnormal myocardium layer morphology cardiac hypertrophy enlarged heart	J:44313	J:162532	J:305	J:159750
21297	ILMN_1228557	0.48737071	3.558987457	0.005055627	0.2526753	-1.896042755	Id2					
38993	ILMN_2810937	-0.486909915	-6.062821508	0.000113302	0.0988892	1.477633682	Slc25a42					
4546	ILMN_2513378	0.486813281	4.017102892	0.002368482	0.2115757	-1.204123396	6430537F04					
44455	ILMN_1230396	-0.483151468	-4.182434489	0.001812826	0.2030807	-0.961409236	Zc3h6					
31291	ILMN_2604333	0.482372742	4.337823895	0.001414675	0.1940342	-0.737092515	Npr3					
33818	ILMN_2754377	0.481319247	5.345132855	0.000307726	0.117837	0.619034096	Pde1a					
29248	ILMN_2981545	0.481275894	6.599353309	5.62E-005	0.0988892	2.062588952	Mfap2					
24088	ILMN_1236869	-0.478653762	-4.042436256	0.002272892	0.2114221	-1.166671808	LOC100046690					
39998	ILMN_2665535	0.478392849	4.524657424	0.001054538	0.1832233	-0.472507112	Spon1					
44165	ILMN_2472917	0.47819526	3.741368235	0.003727538	0.2290185	-1.617458875	Wnt5a					
16357	ILMN_2917471	0.478146503	5.28429632	0.000336039	0.117837	0.542130154	Ednrb	pericardial effusion abnormal direction of heart looping enlarged pericardium hemopericardium		J:46640	J:149893	
14879	ILMN_1241225	0.475888407	4.129515774	0.001974028	0.2069119	-1.03865332	Dctd					
15802	ILMN_3009243	0.475459138	4.378615363	0.001326234	0.1909008	-0.678840456	Dusp2					
1752	ILMN_2542320	0.474631036	3.295733589	0.007894664	0.286779	-2.30399852	2410018L13Rik					
42085	ILMN_1250011	-0.474102149	-4.177227793	0.001828055	0.2030807	-0.968990449	Tob1					
35473	ILMN_2649456	0.473581317	3.554019557	0.00509802	0.2530329	-1.903682138	Prr15					
15745	ILMN_1221805	-0.473312739	-3.716175609	0.003886964	0.23405	-1.655712227	Dtna	cardiac fibrosis myocardial fiber degeneration		J:59675		
21595	ILMN_2804166	0.471480348	3.889869099	0.002916455	0.2215772	-1.393588399	Igsf9					
18654	ILMN_2939666	0.469974304	3.213453136	0.009085501	0.2972547	-2.432601106	Fzd2	abnormal heart morphology double outlet heart right ventricle overriding aorta ventricular septal defect		J:165556		
17210	ILMN_2782964	0.469881233	3.64265237	0.004394072	0.2462475	-1.767780517	Enpp1					
34157	ILMN_2882079	0.469139947	3.826017854	0.003239948	0.2239818	-1.489498817	Phf13					
41297	ILMN_1253854	0.46745014	3.507232647	0.005515788	0.2580919	-1.975750975	Tgif1					
33904	ILMN_1235230	0.464064376	3.326198253	0.007495483	0.2821829	-2.256496262	Pdlim3	abnormal heart position or orientation abnormal heart right ventricle morphology abnormal heart right ventricle outflow tract morphology cardiac fibrosis dilated cardiomyopathy dilated heart left ventricle dilated heart right ventricle thin interventricular septum thin ventricular wall trabecula carnea hypoplasia		J:69097		
15919	ILMN_1248267	-0.463161238	-3.656550717	0.004293192	0.2454503	-1.746548137	E030030I06Rik					
10308	ILMN_2895991	-0.462890958	-4.234197461	0.001668487	0.1989607	-0.886266331	Brp44					
38581	ILMN_2942551	-0.462477356	-5.574295909	0.00022193	0.1138323	0.902896888	Sh3rf2					
8494	ILMN_2836607	-0.46154993	-3.347130551	0.007233282	0.2783454	-2.223896524	Art3					
14	ILMN_1233188	0.460852645	5.393507057	0.000287032	0.117837	0.679720714	0610007N19Rik					
11404	ILMN_1247775	-0.460676707	-3.333504127	0.007402876	0.2811013	-2.245114463	Casq2	abnormal myocardial fiber morphology cardiac hypertrophy enlarged heart atrium heart left ventricle hypertrophy increased heart weight thick ventricular wall cardiac hypertrophy enlarged heart atrium heart left ventricle hypertrophy		J:151367	J:124211	
34569	ILMN_3109360	-0.459434639	-5.750275811	0.000173536	0.1020503	1.114665874	Plec1					
2592	ILMN_2622227	-0.459275672	-3.194704867	0.009381695	0.2996383	-2.461960288	4632404M16Rik					
31086	ILMN_1244161	0.459016588	4.379706442	0.00132395	0.1909008	-0.677286027	Nme4					
16094	ILMN_1253191	-0.458810583	-3.527393821	0.005331582	0.2555307	-1.944668658	E230024B12Rik					
38336	ILMN_3001827	-0.457874969	-3.183198161	0.009568358	0.302271	-2.47998866	Serpinf2					
41282	ILMN_2834379	0.457444338	3.463876625	0.005934551	0.263656	-2.042725076	Tgfbi					
38351	ILMN_3136744	-0.457079411	-7.25854764	2.49E-005	0.0941113	2.718755367	Sesn1					
38605	ILMN_2858666	0.456777181	4.969485222	0.000534409	0.135947	0.133797675	Shd					
35735	ILMN_2724942	0.456190331	3.90463599	0.002846562	0.2210895	-1.371484427	Ptgis					
18217	ILMN_1225071	0.455982672	3.631528167	0.004476589	0.2475024	-1.784790499	Fgfr2	heart hypoplasia thin ventricular wall dilated heart atrium abnormal conotruncal ridge morphology abnormal heart development abnormal heart ventricle morphology abnormal interventricular groove morphology abnormal left posterior bundle morphology abnormal trabecula carnea morphology atrium hypoplasia conotruncal ridge hypoplasia double outlet heart right ventricle muscular ventricular septal defect overriding aorta perimembraneous ventricular septal defect thin ventricular wall				
43745	ILMN_2491589	0.455785954	8.541809624	5.91E-006	0.06686	3.819030759	Vgll4					
41784	ILMN_2892650	-0.454000007	-3.471447421	0.005859125	0.2632014	-2.031017385	Tmem35					
38353	ILMN_2654074	-0.453779786	-3.810561358	0.003323748	0.2245934	-1.512795924	Sesn1					
30320	ILMN_1252533	0.453741061	3.519315829	0.005404612	0.2565264	-1.957117629	Myla					
37353	ILMN_2880346	0.453342092	4.002627806	0.002424983	0.2115757	-1.225564009	Rrp1b					
35756	ILMN_2638114	0.452815706	5.685850521	0.000189793	0.1074252	1.037761816	Ptn					
41228	ILMN_2599470	-0.450129034	-3.209177099	0.009152202	0.2979254	-2.439295553	Tex11					

42189	ILMN_2440066	-0.450025971	-3.5830426	0.004855463	0.2497766	-1.859088255	Tpra40				
10189	ILMN_1215252	0.450005483	4.164875276	0.001864724	0.2032446	-0.98699288	Bmp4	perimembraneous ventricular septal defect abnormal heart looping abnormal atrioventricular cushion morphology common atrioventricular valve complete atrioventricular septal defect double outlet heart right ventricle ostium primum atrial septal defect partial atrioventricular septal defect abnormal heart and great artery attachment conotruncal ridge hypoplasia abnormal conotruncal ridge morphology abnormal heart development abnormal semilunar valve morphology aortopulmonary window pericardial effusion perimembraneous ventricular septal defect persistent truncus arteriosis abnormal heart right ventricle morphology abnormal heart ventricle outflow tract morphology thick myocardium heart right ventricle hypertrophy perimembraneous ventricular septal defect atrial septal defect		J:108516 J:79391 J:86001 J:169047 J:89237	J:169047 J:112335 J:108516 J:86001
12281	ILMN_2625893	-0.449884632	-3.89733027	0.002880919	0.2213571	-1.382416391	Ces3				
30585	ILMN_2722864	-0.448839793	-3.249680886	0.008539989	0.2925108	-2.375926118	Ncam1				
30305	ILMN_2978040	-0.448453379	-4.393613266	0.0012952	0.1909008	-0.657490237	Myh8				
33905	ILMN_2885990	0.44765406	4.300971863	0.001499928	0.1946082	-0.789947851	Pdlim3	abnormal heart position or orientation abnormal heart right ventricle morphology abnormal heart right ventricle outflow tract morphology cardiac fibrosis dilated cardiomyopathy dilated heart left ventricle dilated heart right ventricle thin interventricular septum thin ventricular wall trabecula carnea hypoplasia		J:69097	
6846	ILMN_2956932	-0.447524061	-3.312502822	0.007672304	0.2854639	-2.277842814	Acadvl	Abnormal heart morphology; abnormal myocardial fiber morphology;<br>myocardial fiber degeneration; abnormal myocardial fiber morphology		J:72193 J:98627 J:106714	
39494	ILMN_1225376	0.447393841	3.875640413	0.002985499	0.2228076	-1.414914473	Smoc1				
16358	ILMN_2589640	0.447190908	3.812605294	0.003312538	0.2245934	-1.5097134	Ednrb	pericardial effusion abnormal direction of heart looping enlarged pericardium hemopericardium		J:46640 J:149893	
27864	ILMN_2772691	0.447062806	5.752783119	0.000172934	0.1020503	1.117644323	LOC666036				
24420	ILMN_2750740	0.446020301	5.187971977	0.000386709	0.1220452	0.419036592	LOC100048721				
33482	ILMN_2709167	-0.445850009	-4.561415563	0.000995881	0.1789736	-0.42112927	Papln				
44486	ILMN_2437173	0.445152753	3.278455305	0.008130703	0.28763	-2.330968404	Zcchc3				
13748	ILMN_2874352	0.444674385	6.049513081	0.000115346	0.0988892	1.462517132	Cyp17a1				
7835	ILMN_2648397	0.443810996	4.626969085	0.000899678	0.1771726	-0.330065723	Anapc5				
35883	ILMN_2662802	0.442874613	3.353226309	0.007158714	0.2779162	-2.214409273	Ptx3				
6902	ILMN_2799351	-0.442203103	-4.719939747	0.00077977	0.1657689	-0.202163247	Acot11				
4844	ILMN_2675719	-0.442013848	-6.001495641	0.000123058	0.0990647	1.407726992	9030612E09Rik				
33993	ILMN_2813487	-0.441946238	-5.533798166	0.000235	0.1138323	0.853400703	Per1				
21303	ILMN_1243499	0.441442882	4.211138034	0.001731234	0.2015219	-0.919690029	Ide				
42875	ILMN_1236188	0.441141916	6.485235572	6.50E-005	0.0988892	1.942111479	Twist1				
5096	ILMN_2797584	0.440923692	3.170396427	0.009780499	0.3037522	-2.500053938	9430028L06Rik				
9490	ILMN_2720083	0.440867831	4.048665044	0.002250014	0.2114221	-1.157477685	Bace2				
11968	ILMN_1242724	-0.437509265	-3.988890769	0.00247991	0.2130793	-1.245939013	Cdc37l1				
36540	ILMN_2723718	0.435748484	4.975609923	0.000529535	0.135947	0.1419074	Rcn3				
44711	ILMN_2441785	0.435243996	3.288547777	0.007991963	0.286779	-2.315212496	Zfp286				
6575	ILMN_2533447	-0.434937264	-3.426516159	0.006321714	0.2680276	-2.100576361	AA407270				
296	ILMN_2473692	-0.434628481	-3.607658374	0.004659123	0.2486875	-1.821335931	1110059G02Rik				
40382	ILMN_1253304	-0.434537906	-3.489495603	0.005683286	0.2608203	-2.003128716	Stmn2				
2274	ILMN_2799217	0.434313541	5.38103624	0.000292221	0.117837	0.66411504	2810441K11Rik				
29125	ILMN_1214141	0.432110605	6.483646179	6.52E-005	0.0988892	1.940418708	Med19				
35307	ILMN_3158919	0.431245226	3.655512603	0.004300643	0.2454503	-1.7481333	Prkcz				
15397	ILMN_2632034	0.431034116	3.39697578	0.006646235	0.2725999	-2.146404852	Dnahc11	abnormal heart atrium auricular region morphology abnormal heart atrium morphology left atrial isomerism right atrial isomerism dextrocardia mesocardia abnormal heart morphology		J:4058 J:145068 J:107740	
15747	ILMN_2724339	-0.429939838	-3.405667407	0.006549006	0.2707241	-2.132913112	Dtna	cardiac fibrosis myocardial fiber degeneration		J:59675	
39211	ILMN_2675026	-0.429908763	-5.589051056	0.000217364	0.1138323	0.920859492	Slc4a3	cardiac hypertrophy	J:171021		
28406	ILMN_2608166	0.429231782	4.39689137	0.00128852	0.1909008	-0.652828555	Lrrn4				
17575	ILMN_2805829	0.428811863	3.926382094	0.002746806	0.2182072	-1.338987403	Exosc2				
8164	ILMN_3009225	-0.428625764	-3.29085092	0.007960644	0.286779	-2.311617893	Apoc4				
18236	ILMN_2770386	-0.428110911	-3.755751908	0.003639581	0.2274714	-1.595652803	Fhl2	cardiac hypertrophy		J:131577 J:101174 J:111148	
31389	ILMN_2699052	-0.427795739	-3.843957831	0.00314544	0.2231222	-1.46249718	Nrn1				
8175	ILMN_2916782	-0.427381772	-3.532316878	0.005287577	0.2555307	-1.937084931	Apom				
10359	ILMN_2734097	-0.427358421	-3.404031083	0.006567198	0.2707241	-2.135452643	Btc	abnormal atrioventricular valve morphology abnormal semilunar valve morphology cardiac fibrosis dilated heart left atrium enlarged aortic valve enlarged pulmonary valve		J:83820	
30408	ILMN_1257860	-0.425718616	-3.375352373	0.006894632	0.2750624	-2.179997233	Myoz2	abnormal myocardial fiber morphology cardiac hypertrophy dilated heart atrium dilated heart left ventricle dilated heart right ventricle enlarged heart small myocardial fiber		J:94665	
22332	ILMN_2765662	0.425648568	5.249304515	0.000353575	0.117837	0.497602336	Kcne1l				
44560	ILMN_2763951	0.425574475	6.136849754	0.000102619	0.0988892	1.561175271	Zfand2b				
14305	ILMN_2579391	-0.424897306	-5.162118528	0.000401656	0.1220628	0.385720944	D330026F23Rik				

37656	ILMN_2609421	-0.423005679	-3.636531065	0.004439282	0.2471922	-1.777138875	Scgb1c1					
11303	ILMN_2695143	0.422894923	4.523655448	0.001056187	0.1832233	-0.473910755	Capn6					
39713	ILMN_1255551	-0.421401373	-4.390729076	0.001301108	0.1909008	-0.661593188	Sorbs1					
37192	ILMN_1227653	0.420456369	4.005088974	0.002415277	0.2115757	-1.221916397	Rpl27a					
12436	ILMN_2740151	-0.419472611	-4.025082089	0.002337927	0.2115757	-1.192317374	Chpt1					
35073	ILMN_2651660	-0.418637224	-4.463268859	0.001160792	0.1848813	-0.558812192	Ppp1r1b					
29175	ILMN_2850391	0.418018398	4.509642448	0.00107954	0.1836985	-0.493558718	Meis2					
17508	ILMN_3005058	-0.417900919	-4.198427359	0.001766878	0.2023173	-0.938148759	Etfdh					
3884	ILMN_2944601	0.417526146	3.224590109	0.008914103	0.2952141	-2.415170182	4933439C20Rik					
18314	ILMN_2926842	0.416707819	4.0749002	0.002156272	0.2114221	-1.118814845	Flrt2	abnormal endocardium morphology abnormal epicardium morphology pericardial edema ventricular myocardium compact layer hypoplasia		J:171515		
9971	ILMN_2765224	-0.416527209	-3.225012761	0.008907664	0.2952141	-2.414508813	Bcam					
43248	ILMN_2461668	0.415683877	3.850424356	0.003112084	0.2231222	-1.452774641	Unc5c					
36185	ILMN_1239542	0.415313428	5.877098287	0.000145734	0.1020503	1.263961572	Raet1c					
1303	ILMN_2522062	0.415234273	4.582461769	0.00096386	0.1789736	-0.391814194	2010204K13Rik					
18557	ILMN_1216722	-0.414443322	-4.430433821	0.001222216	0.1878937	-0.605229384	Ftcd					
29073	ILMN_3080012	0.414429139	5.509092521	0.000243375	0.1138323	0.823064957	Mdk					
7567	ILMN_1219231	-0.413579299	-4.076802348	0.002149637	0.2114221	-1.116015562	Akap1					
33815	ILMN_3146952	0.413405868	3.292198876	0.007942372	0.286779	-2.309514255	Pde1a					
6916	ILMN_2807084	0.413044032	4.323554114	0.001447065	0.1946082	-0.757533401	Acot7					
3576	ILMN_1214466	-0.412628538	-4.590796037	0.000951482	0.1789736	-0.380226022	4932408C11Rik					
16297	ILMN_2745993	0.412434432	3.358136472	0.007099224	0.2773512	-2.206769325	Ece1	abnormal atrioventricular valve morphology abnormal heart valve morphology double outlet heart right ventricle failure of atrioventricular cushion closure perimembraneous ventricular septal defect persistent truncus arteriosis double outlet heart right ventricle overriding aorta persistent truncus arteriosis abnormal heart and great artery attachment double outlet heart right ventricle overriding aorta perimembraneous ventricular septal defect persistent truncus arteriosis decreased atrioventricular cushion size double outlet heart right ventricle enlarged heart overriding aorta pericardial effusion ventricular septal defect		J:62261	J:48566	J:46640
38141	ILMN_2684289	0.411791371	3.477952699	0.005795107	0.2619621	-2.020961685	Sema3a	dilated heart right atrium dilated heart right ventricle heart right ventricle hypertrophy	J:35824			
5097	ILMN_2510128	0.411513978	3.633739114	0.004460062	0.2471922	-1.781408649	9430028L06Rik					
21794	ILMN_2724643	0.411072023	4.157083236	0.001888252	0.2032446	-0.998360855	Imp4					
24122	ILMN_2679037	0.409525928	4.606300042	0.000928902	0.1785243	-0.358699967	LOC100046883					
3920	ILMN_2704184	0.408933585	3.992541744	0.002465186	0.2130793	-1.240521214	5031439A09Rik					
391	ILMN_2632839	0.408903231	3.630321441	0.004485636	0.2477001	-1.786636529	1300007L22Rik					
19059	ILMN_2895557	-0.408650716	-3.251627884	0.00851164	0.2923062	-2.37288244	Gfi1b					
33994	ILMN_2813484	-0.408442868	-3.607391992	0.004661203	0.2486875	-1.821744123	Per1					
42907	ILMN_1250947	0.407412807	6.15091684	0.000100714	0.0988892	1.576946091	Txndc5					
9240	ILMN_1252723	-0.40736393	-3.289171764	0.007983465	0.286779	-2.314238581	B230365C01Rik					
20002	ILMN_2666181	-0.407180977	-4.243499717	0.001643857	0.1989607	-0.872806458	Grb14	increased heart weight		J:87892		
40376	ILMN_2794051	0.40697921	5.824756132	0.000156581	0.1020503	1.202679431	Stk38l					
18645	ILMN_2750912	-0.405875724	-4.971100615	0.000533119	0.135947	0.135937253	Fyco1					
2535	ILMN_1245109	0.405685742	3.482822622	0.005747655	0.2616355	-2.013436468	3830430K15Rik					
18117	ILMN_1229519	-0.405493127	-3.945748786	0.002661042	0.2182072	-1.310100686	Fech					
7387	ILMN_2972521	-0.405426084	-6.184746494	9.63E-005	0.0988892	1.614737521	Agtr1a	abnormal heart left ventricle morphology cardiac fibrosis cardiac interstitial fibrosis increased heart weight decreased heart weight muscular ventricular septal defect perimembraneous ventricular septal defect ventricular septal defect decreased heart weight decreased heart weight decreased heart weight decreased heart weight decreased heart weight decreased heart weight		J:124206	J:46007	J:137944
22754	ILMN_2946653	0.404273587	4.369650949	0.001345157	0.1909407	-0.691619083	Klk1b22					
11514	ILMN_2944226	0.40407046	3.886733682	0.002931525	0.2219772	-1.39828546	Ccdc124					
13150	ILMN_3083163	-0.402889702	-3.690996182	0.004053408	0.2381602	-1.694021331	Cp					
21298	ILMN_2887239	0.402833207	3.175994989	0.009687138	0.3028931	-2.49127782	Id2					
33870	ILMN_2753912	0.402415236	5.090301338	0.000446499	0.12825	0.29255936	Pdgfc	absent atrial septum pericardial effusion		J:93954		
33997	ILMN_2987863	-0.402137564	-3.926942506	0.002744284	0.2182072	-1.338150786	Per2					
17174	ILMN_2598103	-0.402114221	-3.799451536	0.003385376	0.2245934	-1.529560325	Emp2					
24421	ILMN_2756578	0.401973877	3.841350402	0.003158995	0.2231222	-1.466419044	LOC100048721					
29482	ILMN_2754447	0.401731015	3.502293876	0.005561907	0.2585716	-1.983371098	Mkrn3					
19142	ILMN_2547942	0.400908479	4.818123972	0.00067134	0.1519947	-0.068693731	Gins2					
23858	ILMN_1249684	0.400302379	4.429452573	0.001224104	0.1878937	-0.606619241	LOC100045019					
9640	ILMN_1226639	-0.400122269	-4.041673885	0.002275708	0.2114221	-1.167797506	BC006779					

Supplementary Table 1

To identify perturbed genes and molecular pathways that could explain the phenotypic effects of Ddc_exon1a ablation in the heart, changes in gene expression in $Ddc^{PAT\Delta}$ hearts were assayed using the Illumina™ WG6 expression microarray. The Illumina WG-6 mouse microarray platform assayed 45281 probes in biological replicates for three DdcWT, four $Ddc^{MAT\Delta}$, four $Ddc^{PAT\Delta}$ and one $Ddc^{\Delta\Delta}$ heart. Gene expression in $Ddc^{PAT\Delta}$ animals was compared to $Ddc^{MAT\Delta}$. The probe IDs on the array are shown with gene names, log fold change and p value and adjusted p value along with annotations of cardiovascular related genes with differential expression. To assess differential expression at each probe, a linear modelling approach that modelled genotype as a factor compared $Ddc^{MAT\Delta}$, mice that have near wildtype cardiac Ddc_exon1a expression and $Ddc^{PAT\Delta}$, mice that exhibit reduced cardiac Ddc_exon1a expression. Only Ddc itself was significantly different between genotypes after correction for multiple testing. An approach that combined a biologically plausible change in transcript expression equivalent to an absolute log2 fold-change of >0.4 (representing an approximately 30% increase or 25% decrease in gene expression) and a statistical significance threshold of $P < 0.01$ was also used.
