# Supplementary material for: Imprinted Gene Expression and Function of the Dopa Decarboxylase Gene in the Developing Heart
Source: Front Cell Dev Biol. 2021 Jun 22;9:676543. doi: 10.3389/fcell.2021.676543 (PMC8258389; doi:10.3389/fcell.2021.676543)
Supplement: Supplementary file 6 [file Data_Sheet_1.PDF]

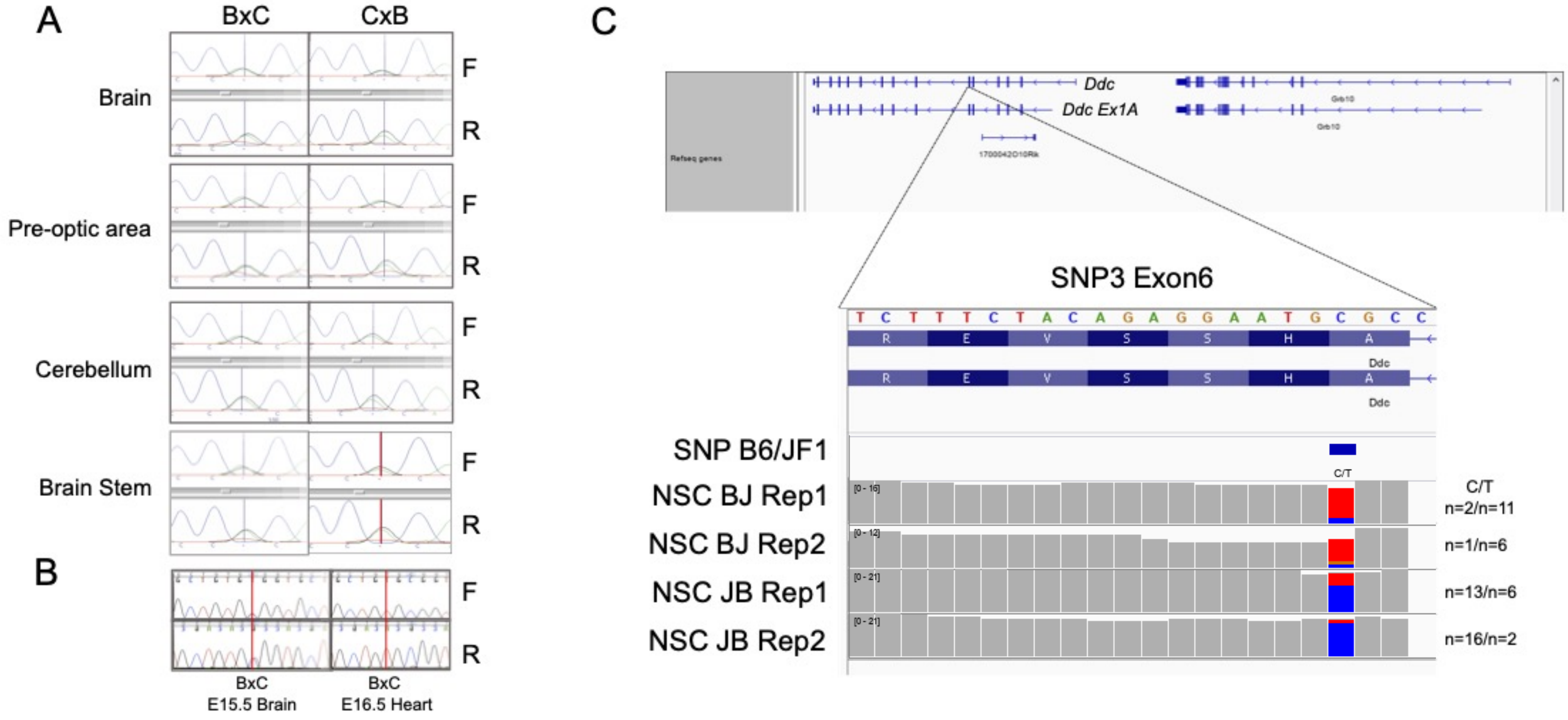

D

	SNP1 (Ex2)			SNP2 (Ex3)			SNP3 (Ex6)			SNP4 (Ex8)			SNP5 (Ex15)			SNP6 (Ex15)		
	C (Bl6)	T (JF1)	Allelism	A (Bl6)	G (JF1)	Allelism	C (Bl6)	T (JF1)	Allelism	C (Bl6)	T (JF1)	Allelism	T (Bl6)	C (JF1)	Allelism	T (Bl6)	C (JF1)	Allelism
NSC BJ Rep1	0	8	Pat (100%)	0	7	Pat (100%)	2	11	Pat (85%)	1	7	Pat (87%)	0	10	Pat (100%)	0	5	Pat (100%)
NSC BJ Rep2	0	2	Pat (100%)	0	4	Pat (100%)	1	6	Pat (86%)	2	11	Pat (78%)	0	5	Pat (100%)	0	3	Pat (100%)
NSC JB Rep1	4	8	Mat (67%)	14	6	Pat (70%)	13	6	Pat (81%)	5	3	Pat (62%)	5	2	Pat (71%)	2	1	Pat (67%)
NSC JB Rep2	5	0	Pat (100%)	4	1	Pat (80%)	16	2	Pat (89%)	8	0	Pat (100%)	13	7	Pat (65%)	1	1	
