# Supplementary figures and images for: Imprinted Gene Expression and Function of the Dopa Decarboxylase Gene in the Developing Heart
Source: Front Cell Dev Biol. 2021 Jun 22;9:676543. doi: 10.3389/fcell.2021.676543 (PMC8258389; doi:10.3389/fcell.2021.676543)

Ddc

BxC

Brain

Heart

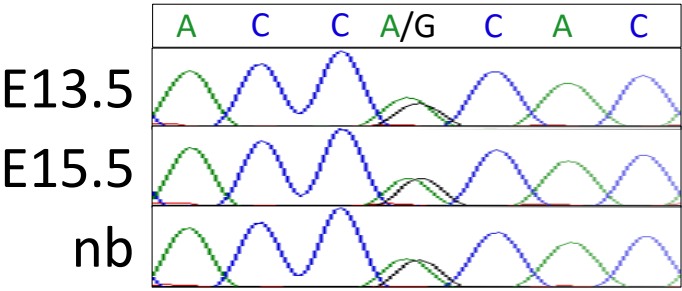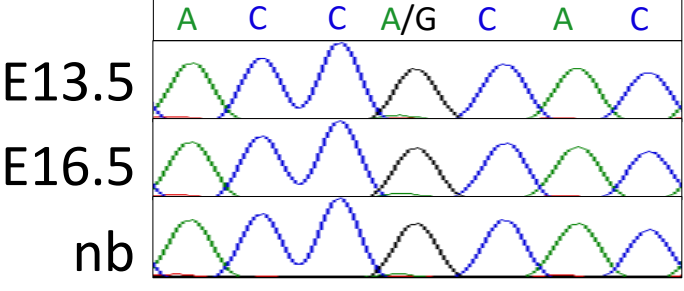

AK006690

BxC

Brain

Heart

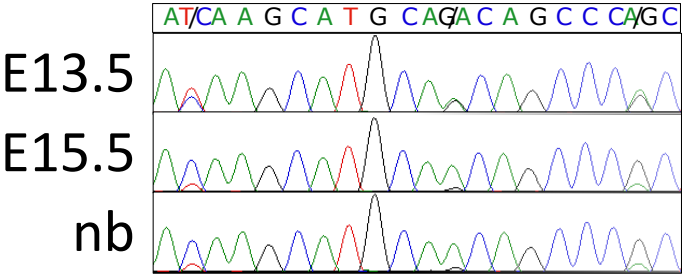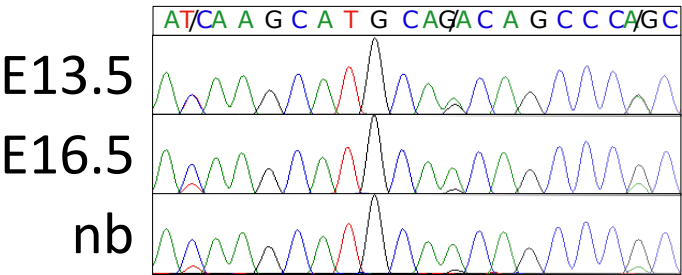

CxB

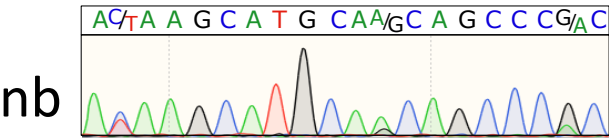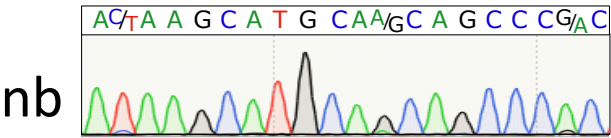

Supplement: Supplementary file 7 [file Data_Sheet_2.PDF]

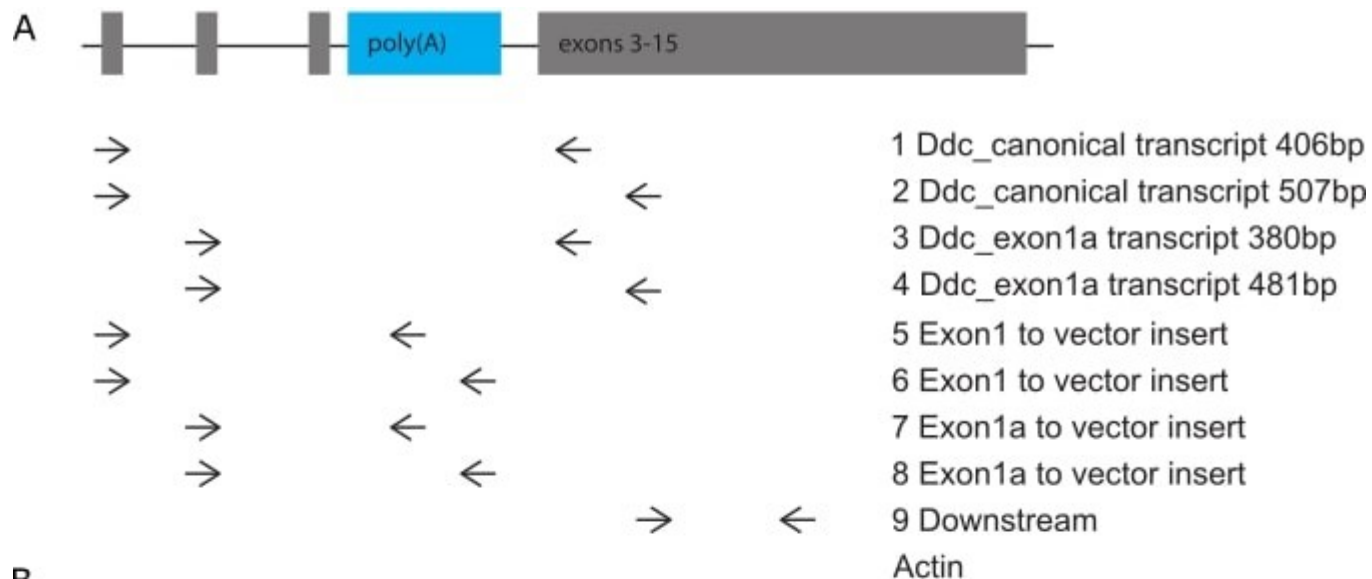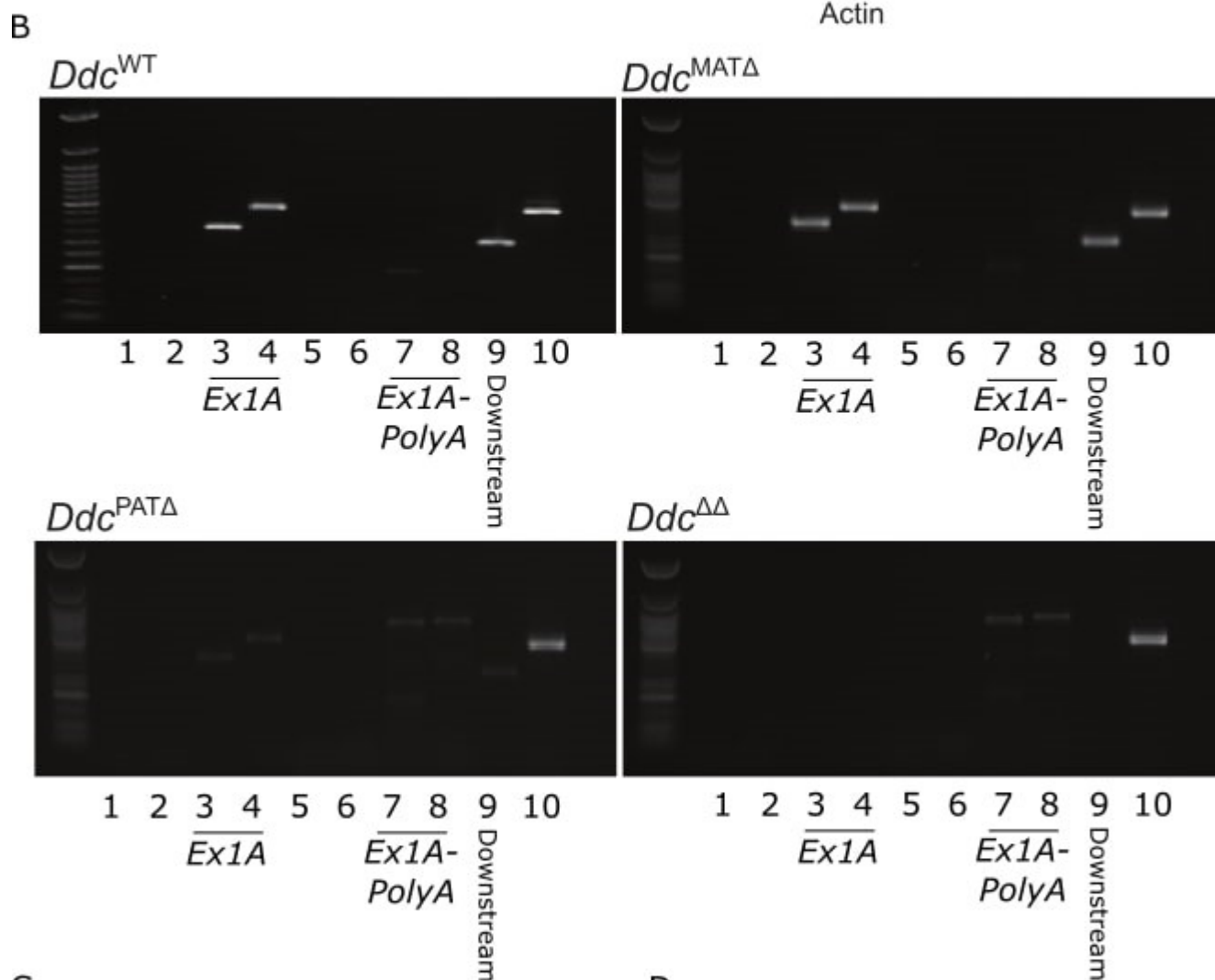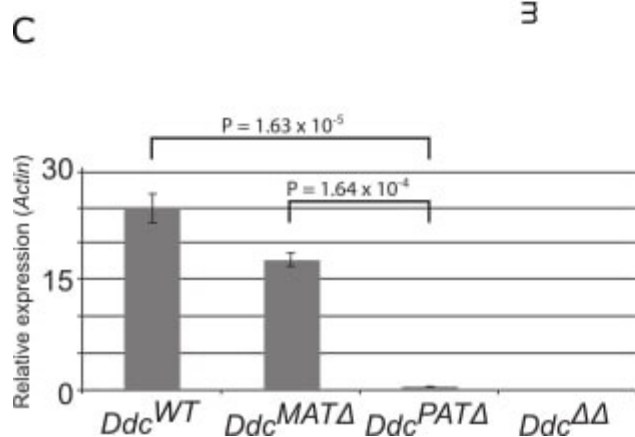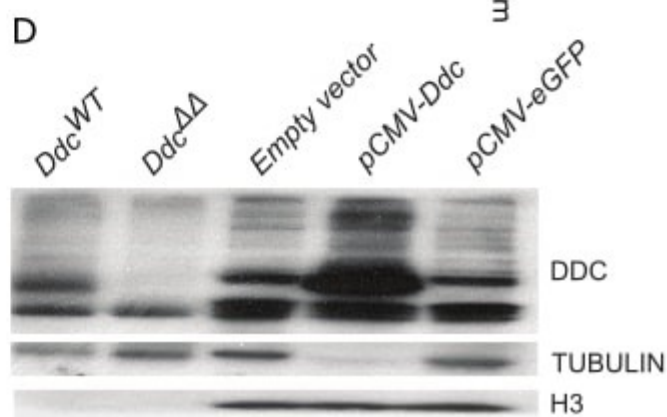

Supplement: Supplementary file 8 [file Data_Sheet_3.PDF]
