# Supplementary material for: Imprinted Gene Expression and Function of the Dopa Decarboxylase Gene in the Developing Heart
Source: Front Cell Dev Biol. 2021 Jun 22;9:676543. doi: 10.3389/fcell.2021.676543 (PMC8258389; doi:10.3389/fcell.2021.676543)
Supplement: Supplementary file 9 [file Data_Sheet_4.PDF]

Supplementary Figure Legends: Prickett *et al.*

Supplementary Figure 1

Allele-specific assays of brain regions, brain and heart to detect parent-of-origin-specific gene expression of *Ddc_exon1a*. RNA from the brain regions indicated (A), from six-week-old mice from reciprocal crosses (BxC indicates the maternal allele is from C57Bl6 and the paternal from *Mus mus castaneus*, and vice versa for CxB). RT-PCR and Sanger sequencing over regions with known SNPs between strains was used to confirm the imprinting status in the embryonic stage in brain (B) shows BxC E15.5 brain and BxC E16.5 heart. The red vertical bars indicate the SNPs between strains and in brain regions from neonates and E15.5 brain, both alleles are present in roughly equal proportions indicating biallelic expression compared to E16.5 heart where only the single paternally expressed, (imprinted) allele is present. (C) Screenshot from the IGV genome browser (mouse mm9 genome) with the different isoforms found at the locus. A zoom in on one of the SNPs found between C57Bl6 and JF1 strain in *Ddc* shows the number of reads carrying one allele or the other (T in red, C in blue or grey). Two replicates from reciprocal crosses in neural stem cells (NSC) are shown. (D) Summary tables for the six different SNP localised inside *Ddc* transcripts. The allele with the highest number of reads shows a bias toward the paternal allele in neural stem cells.

Supplementary Figure 2

Allele specific assays of Ddc in e13.5, e15.5 and newborn (nb) BxC brain show biallelic expression (2 peaks at the SNP) whereas in heart at e13.5, e16.5 and nb, paternal expression is revealed by a single peak at the SNP. The AK006690 antisense transcript assay illustrates three inter-sub specific SNPs indicated by the backslash between 2 bases). In brain and heart, the transcript is biallelic at E13.5. At the later stages of E15.5 and newborn brain and E16.5 and newborn heart, a paternal bias in expression is detected. In the reciprocal CxB assay in brain, two of the SNPs are biallelic and one has a slight bias suggesting that there is no strong imprinting in brain. In the CxB reciprocal nb heart, there is a swap over of the parental origin of the expressed allele compared to BxC supporting a parental-specific expression bias in this tissue. B= C57Bl6 strain, C= *Mus castaneus* strain.

Supplementary Figure 3

RNA transcript analysis in Ddc knockout mouse heart at e15.5. (A) primer locations are indicated by black arrows, forward primers were designed in exon 1, exon 1a and exon 4. Reverse primers were designed in exon 3, exon 4 and exon 5, plus two pairs in the VICTR48 vector. RT-PCR experiments (lanes 1-10) are indicated to the right of the primer locations. Results are shown in (B) RT-PCR of Ddc^{WT}, $Ddc^{MAT\Delta}$, $Ddc^{PAT\Delta}$ and $Ddc^{\Delta\Delta}$ expression are as expected except in the $Ddc^{PAT\Delta}$ genptype where *Ddc_exon1a* expression is present despite the fact that the maternal allele is epigenetically silenced (lanes 3,4). (C) depicts the results of a quantitative PCR analysis in e15.5 hearts for Ddc transcript in Ddc^{WT}, $Ddc^{MAT\Delta}$, $Ddc^{PAT\Delta}$ and $Ddc^{\Delta\Delta}$ genotypes. $Ddc^{PAT\Delta}$ has a diminished expression of *Ddc_exon1a* as expected. (D) western blot analysis on protein extract from either Ddc^{WT}, or $Ddc^{\Delta\Delta}$ whole carcass and NIH3T3 fibroblast cell lines transfected with either Empty Vector, Ddc under the control of the cauliflower mosaic virus promoter (pCMV Ddc) or eGFP under the control of the cauliflower mosaic virus promoter (pCMV eGFP). The western blot was probed using antibodies raised against DDC, followed by Tubulin and Histone H3 as loading controls. Tubulin detection in the pCMV transfected fibroblast lane is light, possibly because of the high

levels of DDC staining at the same location, the blot was re-probed for histone H3 which stained as expected. The RT-PCR (B) was performed once, qPCR for Ddc expression (C) was repeated 4x (Ddc^{WT}), 3x ($Ddc^{MAT\Delta}$), 3x ($Ddc^{PAT\Delta}$) and 2x ($Ddc^{\Delta\Delta}$) and the western blot (D) was performed once.

Supplementary Tables

Supplementary Table 1

To identify perturbed genes and molecular pathways that could explain the phenotypic effects of Ddc_exon1a ablation in the heart, changes in gene expression in $Ddc^{PAT\Delta}$ hearts were assayed using the Illumina™ WG6 expression microarray. The Illumina WG-6 mouse microarray platform assayed 45281 probes in biological replicates for three Ddc^{WT}, four $Ddc^{MAT\Delta}$, four $Ddc^{PAT\Delta}$ and one $Ddc^{\Delta\Delta}$ heart. Gene expression in $Ddc^{PAT\Delta}$ animals was compared to $Ddc^{MAT\Delta}$. The probe IDs on the array are shown with gene names, log fold change and p value and adjusted p value along with annotations of cardiovascular related genes with differential expression. To assess differential expression at each probe, a linear modelling approach that modelled genotype as a factor compared $Ddc^{MAT\Delta}$, mice that have near wildtype cardiac *Ddc_exon1a* expression and $Ddc^{PAT\Delta}$, mice that exhibit reduced cardiac *Ddc_exon1a* expression. Only *Ddc* itself was significantly different between genotypes after correction for multiple testing. An approach that combined a biologically plausible change in transcript expression equivalent to an absolute log2 fold-change of >0.4 (representing an approximately 30% increase or 25% decrease in gene expression) and a statistical significance threshold of $P < 0.01$ was also used.
